# Data-driven discovery of molecular photoswitches with multioutput Gaussian processes†

**DOI:** 10.1039/d2sc04306h

**Published:** 2022-11-10

**Authors:** Ryan-Rhys Griffiths, Jake L. Greenfield, Aditya R. Thawani, Arian R. Jamasb, Henry B. Moss, Anthony Bourached, Penelope Jones, William McCorkindale, Alexander A. Aldrick, Matthew J. Fuchter, Alpha A. Lee

**Affiliations:** The Cavendish Laboratory, Department of Physics, University of Cambridge Cambridge CB3 0HE UK rrg27@cam.ac.uk aal44@cam.ac.uk; Molecular Sciences Research Hub, Department of Chemistry, Imperial College London London W12 0BZ UK; Center for Nanosystems Chemistry (CNC), Institut für Organische Chemie, Universität Würzburg Würzburg 97074 Germany; The Computer Laboratory, University of Cambridge Cambridge CB3 0FD UK; Secondmind.ai Cambridge CB2 1LA UK; The Institute of Neurology, Department of Neurology, University College London London WC1N 3BG UK

## Abstract

Photoswitchable molecules display two or more isomeric forms that may be accessed using light. Separating the electronic absorption bands of these isomers is key to selectively addressing a specific isomer and achieving high photostationary states whilst overall red-shifting the absorption bands serves to limit material damage due to UV-exposure and increases penetration depth in photopharmacological applications. Engineering these properties into a system through synthetic design however, remains a challenge. Here, we present a data-driven discovery pipeline for molecular photoswitches underpinned by dataset curation and multitask learning with Gaussian processes. In the prediction of electronic transition wavelengths, we demonstrate that a multioutput Gaussian process (MOGP) trained using labels from four photoswitch transition wavelengths yields the strongest predictive performance relative to single-task models as well as operationally outperforming time-dependent density functional theory (TD-DFT) in terms of the wall-clock time for prediction. We validate our proposed approach experimentally by screening a library of commercially available photoswitchable molecules. Through this screen, we identified several motifs that displayed separated electronic absorption bands of their isomers, exhibited red-shifted absorptions, and are suited for information transfer and photopharmacological applications. Our curated dataset, code, as well as all models are made available at https://github.com/Ryan-Rhys/The-Photoswitch-Dataset.

## Abstract 2

We present a data-driven discovery pipeline for molecular photoswitches through multitask learning with Gaussian processes. Through subsequent screening, we identify several motifs with separated and red-shifted electronic absorption bands.

## Graphical abstract

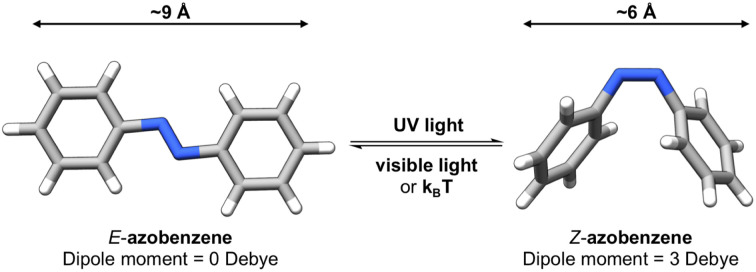

## 1 Introduction

**Fig. 1:**
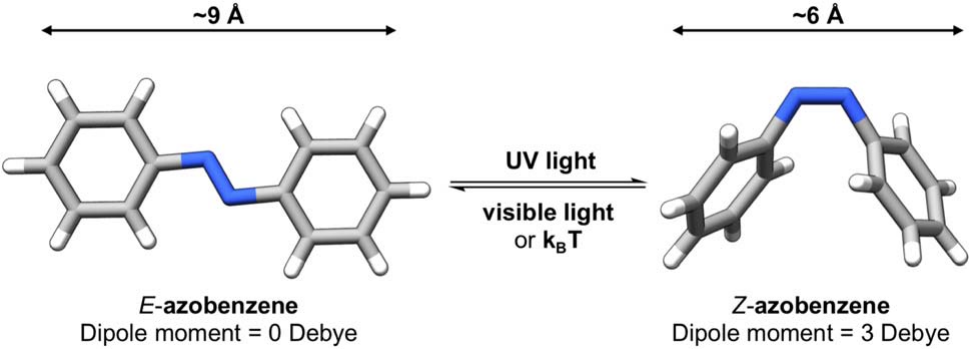
Photoswitchable molecules undergo reversible structural changes between multiple states upon irradiation with light.

Photoswitches^1^ are molecules that can change their structure and properties in response to light as illustrated in Fig. 1. Photoswitches have found increasing use in molecular,^2–5^ supramolecular,^6–8^ and materials applications.^9–13^ On the molecular level, the incorporation of a photoswitchable motif into a drug molecule can provide a means of turning on, or off, its activity using light.^14,15^ Photoswitchable molecules have demonstrated use as the active moiety in light-responsive molecular pumps, serving to drive systems out of equilibrium.^6,16^ Materials designed to transfer information,^17,18^*via* light, have also benefited from the incorporation of photoswitchable molecules as the responsive component. In all of these examples, the structure of the photoswitch,^1,19^ and hence its photophysical properties, is a key consideration to efficient light addressability.

Azobenzene-based photoswitches switch about their N=N bond giving rise to two isomeric forms, *cis*–*trans* or *E*–*Z* isomers. These photoswitches are commonly employed in applications seeking to exploit the significant change in structure, dipole moment, or conductivity of their isomeric forms.^20,21^ Recently, azoheteroarenes, where one or more of the phenyl rings of azobenzene are replaced by heteroarene rings, have emerged as a promising subclass of the azobenzene photoswitch.^18^ Azoheteroarenes demonstrate an expansive structural–property tunability of their photophysical properties. These properties include the degree of photoswitching induced by a specified wavelength, quantified by the photostationary state (PSS), and the thermal half-life of the metastable photogenerated state.

Factors that can determine an azoarene's usefulness in a particular application include the thermal half-life of the metastable isomer, quantum yields of photoswitching and the steady-state distribution of a given isomer at a particular irradiation wavelength (PSS). The ideal thermal half-life is dependent on the targeted application, for example, information transfer requires photoswitches with short thermal half-lives^11^ whilst energy storage applications benefit from photoswitches with long thermal half-lives.^10^ Achieving a high PSS and separated electronic absorption bands of the isomers is generally desirable, however, as these properties determine the addressability of each isomeric form. Through chemical design, the π–π* and n–π* bands of the *E* and *Z* isomers can be tuned to ensure minimal spectral overlap for a given irradiation wavelength, maximising the composition of a specific isomer at said PSS. Moreover, red-shifting the absorption spectra away from the UV region is also beneficial; use of low energy light reduces photo-induced degradation of materials, and also increases the penetration depth in tissue.^5^ Taken together, azoarene photoswitches have been harnessed in a myriad of applications including photopharmacology,^22^ organocatalysis,^23^ molecular solar thermal energy storage,^24,25^ data storage, real-time information transfer,^26^ MRI contrast agents,^27^ and chemical sensing.^28^

To date, structural features that dictate the photophysical properties of these systems are typically post-rationalised following the synthesis and characterisation of a novel structure^19,29–31^ or predicted using quantum chemical calculations such as density functional theory (DFT) and time-dependent density functional theory (TD-DFT).^19,31^ Both of these approaches are limited by the time it takes to perform the synthesis or the calculation *in silico*, although it should be noted that high-throughput DFT approaches may have potential to mitigate the wall-clock time to some extent in the future.^32–34^ In light of this, human intuition remains the guide for candidate selection in many photoswitch chemistry laboratories. Advances in molecular machine learning however, have taken great strides in recent years in areas such as molecule generation,^35–42^ chemical reaction prediction,^43–46^ and molecular property prediction.^47–54^ In particular, machine learning property prediction has the potential to cut the attrition rate in the discovery of novel and impactful molecules by virtue of its short inference time. A rapid, accessible, and accurate machine learning prediction of a photoswitch's properties prior to synthesis would allow promising structures to be prioritised, facilitating photoswitch discovery as well as revealing new structure–property relationships.

Recently work by Lopez and co-workers^55^ employed machine learning to accelerate a quantum chemistry screening workflow for photoswitches. The screening library in this case is generated from 29 known azoarenes and their derivatives yielding a virtual library of 255 991 azoarenes in total. The authors observed that screening using active search tripled the discovery rate of photoswitches compared to random search according to a binary labelling system which assigns a positive label to a molecule possessing a *λ*_max_ > 450 nm and a negative label otherwise. The approach highlights the potential for active learning and Bayesian optimisation methodology to accelerate DFT-based screening. Nonetheless, to our knowledge, the application of machine learning to predict experimental photophysical properties, and the prospective experimental validation of machine learning predictions, remain key open questions.

In this paper we present an experimentally validated framework for molecular photoswitch discovery based on curating a large dataset of experimental photophysical data, and multitask learning using multioutput Gaussian processes. This framework was designed with the goals of: (i) performing faster prediction relative to TD-DFT and directly trained on experimental data; (ii) obtaining improved accuracy relative to human experts; (iii) operationalising model predictions in the context of laboratory synthesis.

To achieve these goals, a dataset of the electronic absorption properties of 405 photoswitches in their *E* and *Z* isomeric forms was curated, a full description of the dataset and collated properties is provided in Section 2. Following an extensive benchmark study, we identified an appropriate machine learning model and molecular representation for prediction, as detailed in Section 3. A key feature of this model is that it is performant in the small data regime as photoswitch properties (data labels) obtained *via* laboratory measurement are expensive to collect in both cost and time. Our model uses a multioutput Gaussian processes (MOGPs) approach due to its ability to operate in the multitask learning setting, amalgamating information obtained from molecules with multiple labels. In Section 4 we show that the MOGP model trained on the curated dataset obtains comparable predictive accuracy to TD-DFT (at the CAM-B3LYP level of theory) and only suffers slight degradations in accuracy relative to TD-DFT methods with data-driven linear corrections whilst maintaining inference time on the order of seconds. A further benchmark against a cohort of human experts as well as a study on how the predictive performance varies as a function of the dataset used for model training is provided in the ESI.† In Section 6 we use our approach to screen a set of commercially available azoarenes, and identify several motifs that display separated electronic absorption bands of their isomers, exhibit red-shifted absorptions, and are suited for information transfer and photopharmacological applications.

## 2 Dataset curation

Experimentally-determined properties of azobenzene-derived photoswitch molecules reported in the literature were curated. We include azobenzene derivatives with a diverse range of substitution patterns and functional groups to cover a large volume of chemical space. This is vitally important from a synthetic point-of-view as such functional groups serve as handles for further synthetic modification. Furthermore, we also included the azoheteroarenes and cyclic azobenzenes which have established themselves as possessing promising photophysical and photochemical properties to unmodified azobenzene motifs.^30^

The dataset includes properties for 405 photoswitches. The molecular structures of these switches are denoted according to the simplified molecular input line entry system (SMILES).^56^ A full list of references for the data sources is provided in Section A of the ESI.† The following properties were collated from the literature, where available. (i) The rate of thermal isomerisation (units = s^−1^), which is a measure of the thermal stability of the metastable isomer in solution. This corresponds to the *Z* isomer for non-cyclic azophotoswitches and the *E* isomer for cyclic azophotoswitches. (ii) The PSS of the stated isomer at the given photoirradiation wavelength. These values are typically obtained by continuous irradiation of the photoswitch in solution until a steady state distribution of the *E* and *Z* isomers is obtained. The reported PSS values correspond to solution-phase measurements performed in the stated solvents. (iii) The irradiation wavelength, reported in nanometers. This corresponds to the specific wavelength of light used to irradiate samples from *E*–*Z* or *Z*–*E* such that a PSS is obtained, in the stated solvent. (iv) The experimental transition wavelengths, reported in nanometers. These values correspond to the wavelength at which the π–π*/n–π* electronic transition has a maximum for the stated isomer. This data was collated from solution-phase experiments in the solvent stated. (v) DFT-Computed Transition Wavelengths, reported in nanometers. These values were obtained using solvent continuum TD-DFT methods and correspond to the predicted π–π*/n–π* electronic transition maximum for the stated isomer. (vi) The extinction coefficient (in units of M^−1^ cm^−1^), corresponding to how strongly a molecular species absorbs light, in the stated solvent. (vii) The theoretically-computed Wiberg Index^57^ (through the analysis of the SCF density calculated at the PBE0/6-31G** level of theory^30^), which is a measure of the bond order of the N=N bond in an azo-based photoswitch, giving an indication of the ‘strength’ of the azo bond.

Using the data collated in this dataset, we focus on using our model to predict the four experimentally-determined transition wavelengths below. We focus on these four properties as they are the core determinants of quantitative, bidirectional photoswitching.^58^ These include, the π–π* transition wavelength of the *E* isomer (data labels for 392 molecules exist in our dataset). The n–π* transition wavelength of the *E* isomer (data labels for 141 molecules exist in our dataset). The π–π* transition wavelength of the *Z* isomer (data labels for 93 molecules exist in our dataset). Finally, the n–π* transition wavelength of the *Z* isomer (data labels for 123 molecules exist in our dataset). We would like to emphasise that other photophysical or thermal properties could also be investigated using machine learning approaches, notably the thermal half-life of the metastable state. However, there are fewer reports of experimentally-derived thermal half-lives significantly reducing the data that we can train our machine learning models on; these other properties will be investigated in future studies.

## 3 Machine learning prediction pipeline

There are three constituents to the prediction pipeline: a dataset, a model and a representation. In terms of the choice of dataset used for model training, we describe our curated dataset in Section 2. We present results in the ESI† comparing models trained on the curated dataset against those trained on a large out-of-domain dataset of 6142 photoswitches.^59^ In terms of the choice of model, we evaluate a broad range including Gaussian processes (GP), random forest (RF), Bayesian neural networks, graph convolutional networks, message-passing neural networks, graph attention networks, LSTMs with augmented SMILES and attentive neural processes (ANP). The full results of our experiments, as well as all hyperparameter settings, are provided in the ESI† where Wilcoxon signed rank tests^60^ determine that there is weak evidence to support that multitask learning affords improvements over the single task setting in the case where auxiliary task labels (*i.e.* not the label being predicted) are available for test molecules. All subsequent experiments in the main paper assume that the MOGP is not provided with auxiliary task labels for test molecules. All experiments may be reproduced *via* the scripts provided at https://github.com/Ryan-Rhys/The-Photoswitch-Dataset. We chose the multioutput Gaussian process (MOGP) to take forward to the comparison against TD-DFT and experimental screening due to its predictive performance in the multitask setting as well as its ability to represent uncertainty estimates. We illustrate some use-cases for uncertainty estimates with confidence–error curves in the ESI.†

An individual box is computed using the mean values of the MAE for the four models for the representation indicated by the associated colour and shows the range in addition to the upper and lower quartiles of the error distribution. The plot indicates that fragprints are the best representation on the *E* isomer π–π* prediction task and RDKit fragments alone are disfavoured across all tasks.

**Fig. 2:**
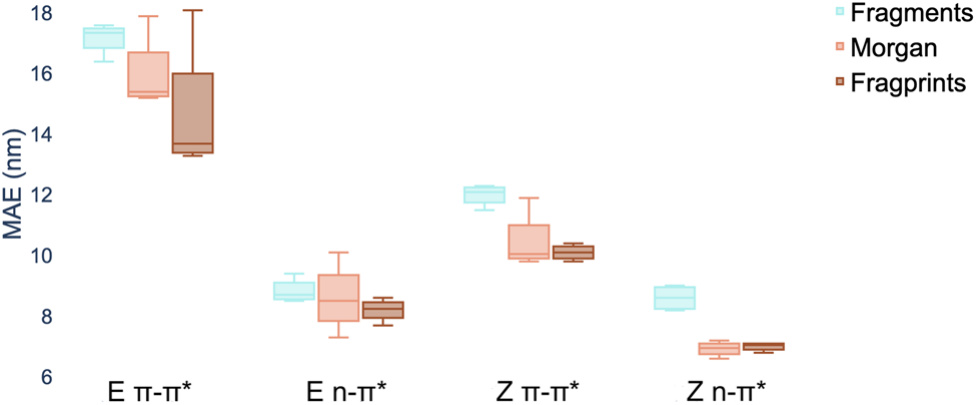
Marginal boxplot showing the performance of representations aggregated over different models (RF, GP, MOGP and ANP). We evaluate performance on 20 random train/test splits of the photoswitch dataset in a ratio of 80/20 using the mean absolute error (MAE) as the performance metric.

In terms of the choice of representation we evaluate three commonly-used descriptors: RDKit fragment features,^61^ ECFP fingerprints^62^ as well as a hybrid ‘fragprints’ representation formed by concatenating the Morgan fingerprint and fragment feature vectors. The performance of the RDKit fragment, ECFP fingerprint and fragprint representations on the wavelength prediction tasks is visualised in Fig. 2 where aggregation is performed over the RF, GP, MOGP and ANP models. This analysis motivated our use of the fragprints representation in conjunction with the MOGP to take forward to the TD-DFT comparison and experimental screening. We now briefly describe Gaussian processes and in particular the multioutput Gaussian process with Tanimoto kernel that we employ for prediction.

### 3.1 Gaussian processes

In the context of machine learning a Gaussian process is a Bayesian nonparametric model for functions. Practical advantages of GPs for molecular datasets include the fact that they have few hyperparameters to tune and maintain uncertainty estimates over property values.^63–65^ A GP is defined as a collection of random variables, {*f*(***x***_**1**_), *f*(***x***_**2**_), …} any finite subset of which are distributed according to a multivariate Gaussian.^63^ A stochastic function 
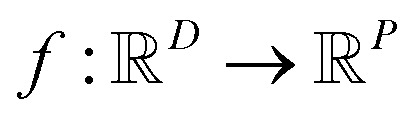
 that follows a GP is fully specified by a mean function *m*(·) and a covariance function or kernel *k*(·, ·) and is written 
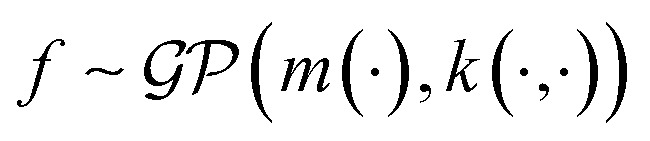
.

When using GPs for molecular property regression tasks we seek to perform Bayesian inference over a latent function *f* that represents the mapping between the inputs {***x***_**1**_, …, ***x***_***N***_} and their property values {*f*(***x***_**1**_), …, *f*(***x***_***N***_)}. In practice we receive the inputs together with potentially noise-corrupted observations of their property values {*y*_1_, …, *y*_*N*_}. The mean function *m*(***x***) is typically set to zero following standardisation of the data. The kernel function *k*(***x***, ***x*′**) computes the similarity between molecules ***x*** and ***x*′**. In all our experiments we use bit/count vectors to represent molecules and hence we choose the Tanimoto kernel^66^ defined as1
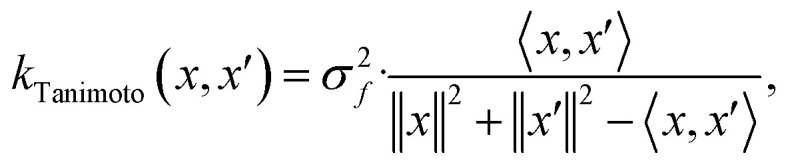
where ***x*** and ***x*′** are count vectors, *σ*_*f*_ is a signal variance hyperparameter and 〈·, ·〉 represents the Euclidean dot product. Given our choice of mean function and kernel we place a GP prior over 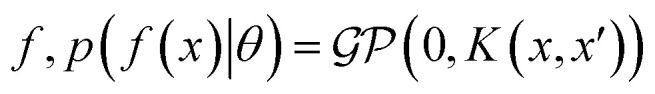 where the notation *K*(***x***, ***x*′**) is taken to mean a kernel matrix whose entries are given as [*K*]_*ij*_ = *k*(***x***_*i*_, ***x***_*j*_) and *θ* as representing the set of kernel hyperparameters (*e.g.* the signal variance in eqn (1)). We also specify a likelihood function *p*(*y*_*i*_|*f*) which depends on *f*(***x***_***i***_) only and is typically taken to be Gaussian 
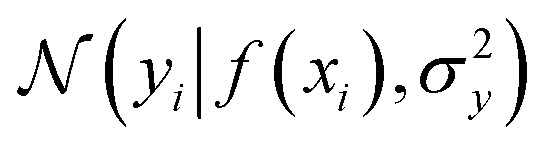
. We assume the noise level *σ*^2^_*y*_ is homoscedastic in this paper but it can also set to be heteroscedastic by introducing a dependence on the input *σ*^2^_*y*_(***x***).^67^ Once we have observed some data (*X*, ***y***), where *X* = {***x***_***i***_}_*i*=1_N is a set of molecules and ***y*** = {*y*_*i*_}_*i*=1_N are their property values, the joint distribution over the observed data *y* and the predicted function values f_*_ at test locations X_*_ may be written as2
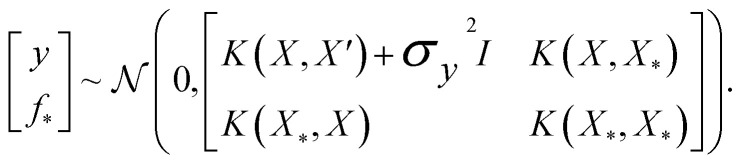


The joint prior in eqn (2) may be conditioned on the observations through 
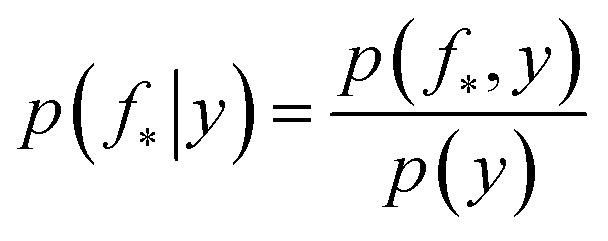
 which enforces that the joint prior agree with the observed target values ***y***. The predictive distribution is given as 
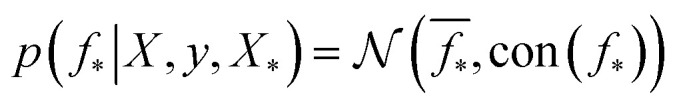
 with the predictive mean at test locations X_*_ being ***f̄***_∗_ = *K*(*X*∗, *X*)[*K*(*X*, *X*) + *σ*^2^_*y*_*I*] − 1***y*** and the predictive uncertainty being cov(***f***_∗_) = *K*(*X*_∗_, *X*_∗_) − *K*(*X*_∗_, *X*)[*K*(*X*, *X*) + *σ*^2^_*y*_*I*]^−1^*K*(*X*, *X*_∗_). The predictive mean is the quantity used for prediction while the predictive uncertainty can inform us as to the model's prediction confidence. The GP hyperparameters are learned through the optimisation of the marginal likelihood where *N* is the number of observations and the subscript notation on the kernel matrix *K*_*θ*_(*X*, *X*′) is chosen to indicate the dependence on the set of hyperparameters *θ*. The two terms in the expression for the marginal likelihood represent the Occam factor^68^ in their preference for selecting models of intermediate capacity. In practical applications, GPs have been primarily employed for their high quality uncertainty estimates across applications 3
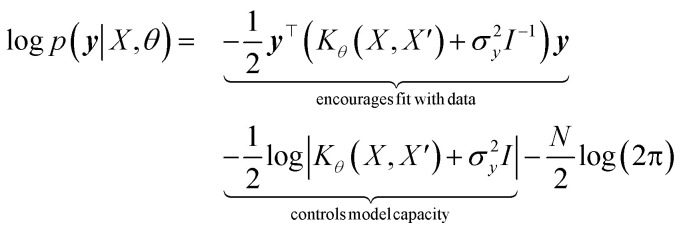
including materials modelling,^69^ astronomical time series modelling,^70^ machine learning hyperparameter tuning,^71,72^ and Bayesian optimisation.^35,73^

### 3.2 Multioutput Gaussian processes (MOGPs)

A MOGP generalises the idea of the GP to multiple outputs and a common use case is multitask learning. In multitask learning, tasks are learned in parallel using a shared representation; the idea being that learning for one task may benefit from the training signals of related tasks. In the context of photoswitches, the tasks constitute the prediction of the four transition wavelengths. We wish to perform Bayesian inference over a stochastic function 
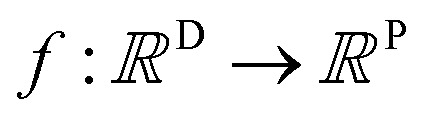
 where *P* is the number of tasks and we possess observations {(***x***_**11**_, *y*_11_), …, (***x***_**1*N***_, *y*_1*N*_), …, (***x***_***P*1**_, *y*_*P*1_), …, (***x***_***PN***_, *y*_*PN*_)}. We do not necessarily have property values for all tasks for a given molecule.

To construct a multioutput GP we compute a new kernel function *k*(***x***, ***x*′**)·*B*[*i*, *j*] where *B* is a positive semidefinite *P* × *P* matrix, where the (*i*, *j*)th entry of the matrix *B* multiplies the covariance of the *i*-th function at ***x*** and the *j*-th function at ***x*′**. Such a multioutput GP is termed the intrinsic model of coregionalisation (ICM).^74^ Inference proceeds in the same manner as for vanilla GPs, substituting the new expression for the kernel into the equations for the predictive mean and variance. Positive semi-definiteness of *B* may be guaranteed through parametrising the Cholesky decomposition LL^T^ where *L* is a lower triangular matrix and the parameters may be learned alongside the kernel hyperparameters through maximising the marginal likelihood in eqn (3) substituting the appropriate kernel. While it has been widely cited that GPs scale poorly to large datasets due to the O(*N*^3^) cost of training, where *N* is the number of datapoints,^63^ recent advances have seen GPs scale to millions of data points using multi GPU parallelisation.^75^ Nonetheless, on CPU hardware scaling GPs to datasets on the order of 10 000 data points can prove challenging. For the applications we consider however, we are unlikely to be fortunate enough to encounter datasets of relevant experimental measurements on the order of tens of thousands of data points and so CPU hardware is sufficient for this study.

## 4 MOGP prediction compared against TD-DFT

We compare the MOGP, Tanimoto kernel and fragprints combination against two widely-utilised levels of TD-DFT: CAM-B3LYP^76^ and PBE0.^77,78^ While the CAM-B3LYP level of theory offers highly accurate predictions, its computational cost is high relative to that of machine learning methods. To obtain the predictions for a single photoswitch molecule one is required to perform a ground state energy minimisation followed by a TD-DFT calculation.^79^ In the case of photoswitches these calculations need to be performed for both molecular isomers and possibly multiple conformations which further increases the wall-clock time. When screening multiple molecules is desirable, this cost, in addition to the expertise required to perform the calculations may be prohibitive, and so in practice it is easier to screen candidates based on human chemical intuition. In contrast, inference in a data-driven model is on the order of seconds but may yield poor results if the training set is out-of-domain relative to the prediction task. Further background on TD-DFT is available in the ESI.†

In Table 1 we present the performance comparison against 99 molecules and 114 molecules for CAM-B3LYP and PBE0 respectively both using the 6-31G** basis set taken from the results of a benchmark quantum chemistry study^80^ to which the reader is referred for all information pertaining to the details of the calculations.§§The TD-DFT CPU runtime estimates are taken from ref. 79 and hence represent a ballpark figure that is liable to decrease with advances in high performance computing. We elect to include an additional 15 molecules in the test set for PBE0. These additional molecules are not featured in the study by Jacquemin *et al.*^80^ but are reported in ref. 30 using the same basis set. It should also be noted that the data presented in Jacquemin *et al.*^80^ contains measurements for the same molecules under different solvents. In our work we absorb solvent effects into the noise. Specifically, we do not treat the solvent as part of the molecular representation. As such, for duplicated molecules we choose a single solvent measurement at random. We report the mean absolute error (MAE) and additionally the mean signed error (MSE) in order to assess systematic deviations in predictive performance for the TD-DFT methods. For the MOGP model, we perform leave-one-out validation, testing on a single molecule and training on the others in addition to the experimentally-determined property values for molecules acquired from synthesis journal papers. We then average the prediction errors and report the standard error.

**Table 1:** MOGP against TD-DFT performance comparison on the PBE0 benchmark consisting of 114 molecules, and the CAM-B3LYP benchmark consisting of 99 molecules. Best metric values for each benchmark are highlighted in bold

Method		Accuracy metric (nm)		CPU runtime (↓)
		MAE (↓)	MSE	
**PBE0 benchmark**				
MOGP		15.5 ± 1.3	**0.0 ± 2.0**	**<1 minute**
PBE0	Uncorrected	26.0 ± 1.8	− 19.1 ± 2.5	
	Linear correction	**12.4** ± **1.3**	− 1.2 ± 1.8	*ca.* 228 days

**CAM-B3LYP benchmark**				
MOGP		15.3 ± 1.4	− 0.2 ± 2.1	**<1 minute**
CAM-B3LYP	Uncorrected	16.5 ± 1.6	6.7 ± 2.2	
	Linear correction	**10.7** ± **1.2**	**0.0 ± 1.6**	*ca.* 396 days

The MOGP model outperforms PBE0 by a large margin and provides comparable performance to CAM-B3LYP. In terms of runtime, there is no contest. The MSE values for the TD-DFT methods however indicate that there is systematic deviation in the TD-DFT predictions. This motivates the addition of a data-driven correction to the TD-DFT predictions. As such, we train a Lasso model with an *L*_1_ multiplier of 0.1 on the prediction errors of the TD-DFT methods and apply this correction when evaluating the TD-DFT methods on the heldout set in leave-one-out validation. We choose to use Lasso as empirically it outperforms linear regression in fitting the errors due to inducing sparsity in the high-dimensional fragprint feature vectors. We show the Spearman rank-order correlation coefficients of all methods and the error distributions in the ESI.† There, it is observed that an improvement is obtained in the correlation between TD-DFT predictions on applying the linear correction. Furthermore, the error distribution becomes more symmetric on applying the correction.

## 5 Human performance benchmark

In practice, candidate screening is undertaken based on the opinion of a human expert due to the speed at which predictions may be obtained. While inference in a data-driven model is comparable to the human approach in terms of speed, we aim in this section to compare the predictive accuracy of the two approaches. In order to achieve this, we assembled a panel of 14 human experts, comprising Postdoctoral Research Assistants and PhD students in photoswitch chemistry with a median research experience of 5 years. The assigned task is to predict the *E* isomer π–π* transition wavelength for five molecules taken from the dataset. A reference molecule is also provided with associated π–π* wavelength. The reference molecule possesses either single, double or triple point changes from the target molecule and serves to mimic the laboratory decision-making process of predicting an unknown molecule's property with respect to a known one.

**Fig. 3:**
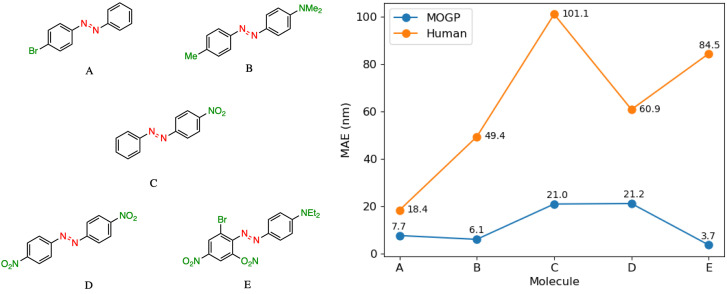
A performance comparison between human experts (orange) and the MOGP-fragprints model (blue). MAEs are computed on a per molecule basis across all human participants.

In all instances, those polled have received formal training in the fundamentals of UV-vis spectroscopy. We note that one of the limitations of this study is that the human chemists are not provided with the full dataset of 405 photoswitch molecules in advance of making their predictions. Our goal in constructing the study in this fashion was to enable a comparison of the benefits of dataset curation, together with a machine learning model to internalise the information contained in the data, against the experience acquired over a photoswitch chemist's research career. Analysing the MAE across all humans per molecule Fig. 3, we note that the humans perform worse than the MOGP model in all instances. In going from molecule A to E, the number of point changes on the molecule increases steadily, thus, increasing the difficulty of prediction. Noticeably, the human performance is approximately five-fold worse on molecule E (three point changes) relative to molecule A (one point change). This highlights the fact that in instances of multiple functional group modifications, human experts are unable to reliably predict the impact on the *E* isomer π–π* transition wavelength. The full results breakdown is provided in the ESI.†

## 6 Screening for novel photoswitches using the MOGP

Having determined that the MOGP approach does not suffer substantial degradation in accuracy relative to TD-DFT we use it to perform experimental screening over 7265 commercially available photoswitch molecules. Diazo-containing compounds supplied by Molport and Mcule were identified. As of November 2020, when the experiments were planned, there were 7265 commercially purchasable diazo molecules. The full list is made available in the GitHub repository. We then used the MOGP to score the list, identifying 11 molecules satisfying our screening criteria detailed in the following section. Our aim is to discover a novel azophotoswitch motif which satisfies the criteria.

### 6.1 Screening criteria

To demonstrate the utility of our approach, we screened commercially available photoswitches based on selective criteria and compared their experimental photophysical properties to the predictions made by the MOGP model. The criteria imposed were selected to showcase that properties could be obtained using the MOGP model which are typically difficult to engineer, yet beneficial for materials and photopharmacological applications. The criteria are:

1. A π–π* maximum in the range of 450–600 nm for the *E* isomer.

2. A separation greater than 40 nm between the π–π* of the *E* isomer and the π–π* of the *Z* isomer.

The first criterion was chosen so as to limit UV-included damage to materials and improve tissue penetration depths and the second criterion was chosen by analogy to azopyrazole photoswitches reported previously^29^ where the specified level of band separation provided complete bidirectional photoswitching; this degree of energetic separation between the π–π* bands of the isomers enables one isomer to be selectively addressed using light emitting diodes (LEDs), which are commonly used for their low power consumption but often express broad emission profiles relative to laser diodes, see ESI.†

### 6.2 Lead candidates

**Fig. 4:**
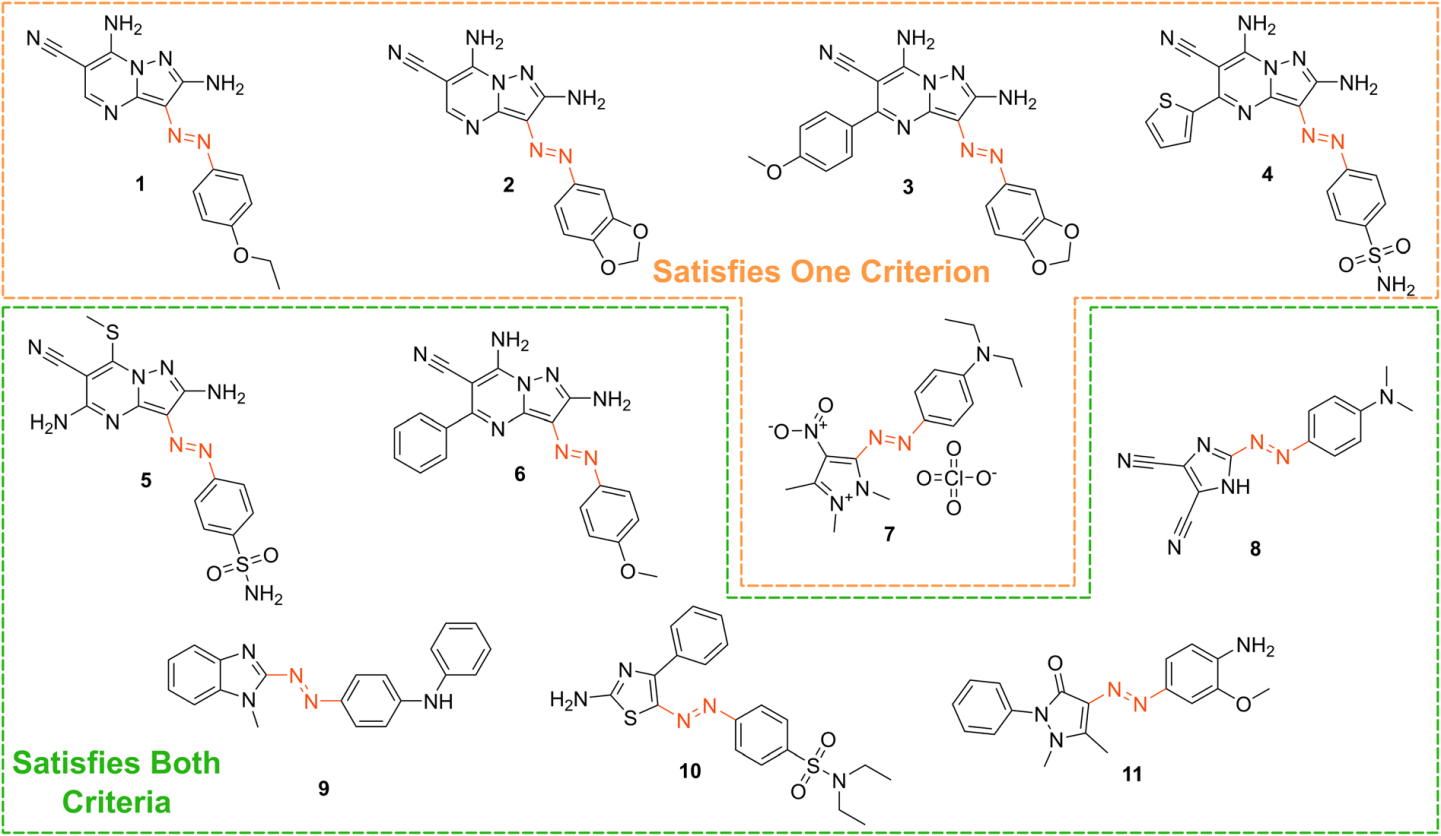
The chemical structures of the 11 commercially available azo-based photoswitches that were predicted to meet the criteria.

**Fig. 5:**
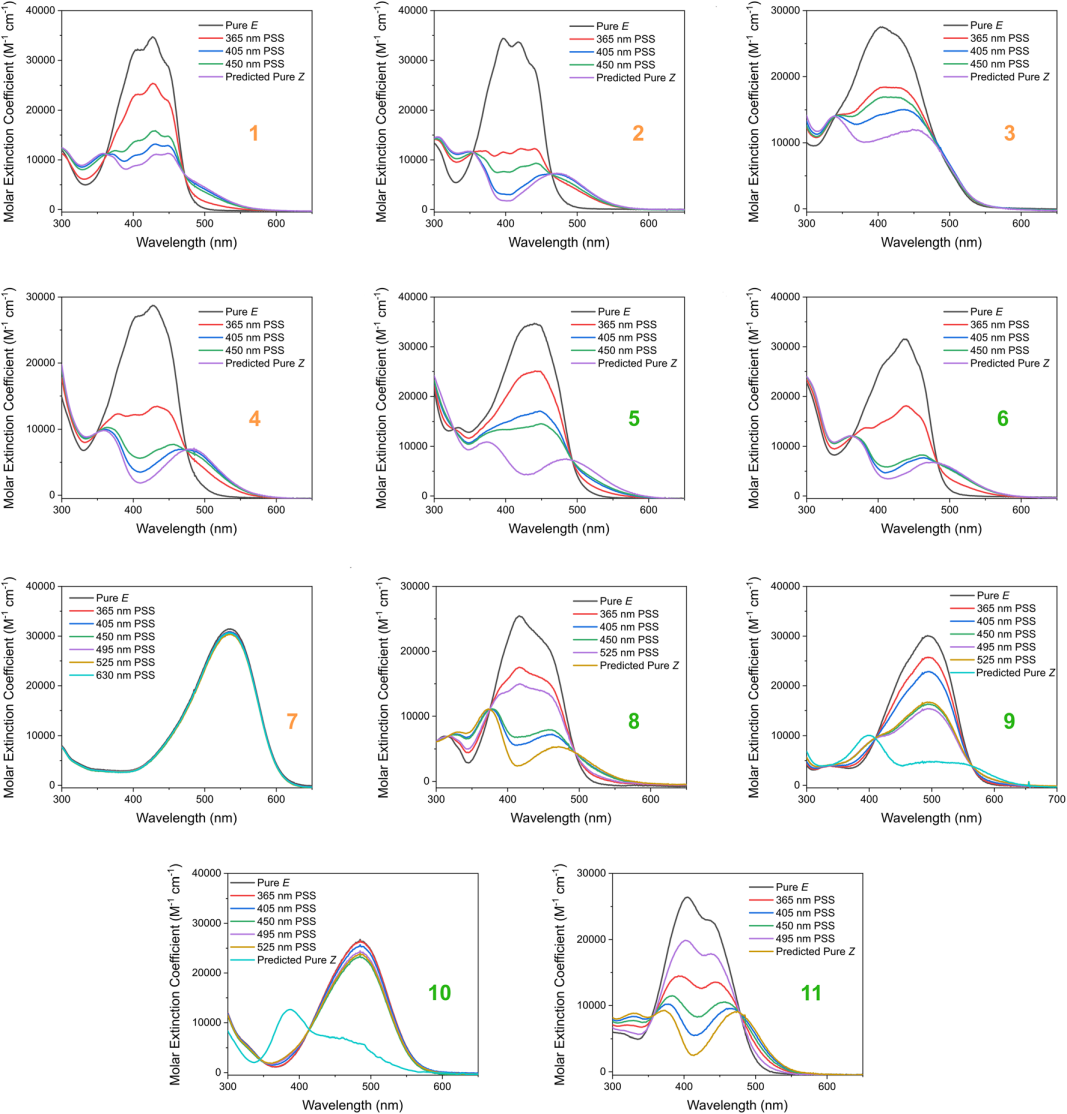
The experimental UV-vis absorption spectrum of switches 1–11 measured at 25 μM in DMSO and shown as the molar extinction coefficient (M^−1^ cm^−1^). Different irradiation wavelengths were employed in order to predict the “pure” *Z* spectra using the procedure detailed by Fischer.^81^ The chemical structures of these switches are shown in Fig. 4 above.

Based on our stated selection criteria, 11 commercially available molecules were identified *via* the predictions of the MOGP model, Fig. 4. The SMILES for these structures are provided in the ESI.† Solutions of these photoswitches were prepared in the dark to a concentration of 25 μM in DMSO. The UV-vis spectra of these photoswitches in their thermodynamically stable *E* isomeric form was recorded using a photodiode array spectrophotometer. The samples were continuously irradiated with various wavelengths of light directed 90° to the measurement path. UV-vis spectra were repeatedly recorded during irradiation until no further change in the UV-vis trace was observed, indicating attainment of the PSS. This *in situ* irradiation procedure was implemented so that even compounds that display short thermal half-lives could be measured reliably. By repeating this measurement process with one or more different irradiation wavelengths, we were able to quantify the PSS and subsequently predict the UV-vis spectrum of the pure *Z* isomer using the method detailed by Fischer.^81^ With both the spectrum of the *E* and *Z* isomers for each photoswitch in hand, the experimentally determined wavelength of the π–π* band of each isomer was determined and compared with that predicted by our model. The spectra are given in Fig. 5. Full experimental details are made available in the ESI.†

We compare the model predictions against the experimentally-determined values in Table 2 The MOGP MAE on the *E* isomer π–π* wavelength prediction task was 22.7 nm and 21.6 nm on the *Z* isomer π–π* wavelength prediction task, comparable for the *E* isomer π–π* and slightly higher for the *Z* isomer π–π* relative to the benchmark study in Section 3, reflecting the challenge of achieving strong generalisation performance when extrapolating to large regions of chemical space. The first criterion, is a requirement on the absolute rather than the relative value of the π–π* transition wavelengths and so the experimental values may be subject to shifts depending on the solvent. Molecules can display solvatochromism in that the dielectric of the solvent, as well as hydrogen-bonding interactions, can influence the electronic transitions giving rise to hypsochromic or bathochromic shifts in the absorption spectra. This can manifest as changes in the position, intensity and shape of the UV-vis absorption spectrum. As such, the 450 nm criterion could be considered a rough guide and candidates that are just short of the threshold may fulfill the criterion in a different solvent. Nonetheless, given that the MOGP model is trained on just a few hundred data points and is asked to extrapolate to several thousand structures, the accuracy is promising with the advent of further experimental data. In terms of satisfying the pre-specified criteria, 7 of the 11 molecules possessed an *E* isomer π–π* wavelength greater than 450 nm, 10 of the 11 molecules possessed a separation between the *E* and *Z* isomer π–π* wavelengths of greater than 40 nm and 6 of the 11 molecules satisfied both criteria. Compound 7 did not photoswitch under irradiation.

**Table 2:** MOGP predictions compared against experimental values (nm). Traffic light system indicates whether the molecules satisfied the criteria. Both criteria (bold) and one criterion (italic). All molecules satisfied at least one criterion. The model MAE was 22.7 nm for the *E* isomer π–π* and 21.6 nm for the *Z* isomer π–π*

Switch	Model		Experimental			
	*E* π–π*	*Z* π–π*	*E* π–π*	*Z* π–π*	*Z* PSS (%)	*ca. t*_1/2_ (s)
*1*	*456*	*368*	*446*	*355*	*90 (405 nm)*	*<5*
*2*	*459*	*377*	*441*	*356*	*96 (405 nm)*	*<1*
*3*	*457*	*377*	*399*	*331*	*66 (405 nm)*	*<10*
*4*	*463*	*373*	*445*	*357*	*94 (405 nm)*	*<1*
**5**	**471**	**381**	**450**	**370**	**68 (450 nm)**	**<1**
**6**	**460**	**368**	**451**	**360**	**92 (405 nm)**	**<30**
*7*	*467*	*369*	*534*	*n/a*	*n/a*	*n/a*
**8**	**450**	**359**	**465**	**376**	**87 (405 nm)**	**<10**
**9**	**453**	**369**	**468**	**399**	**60 (450 nm)**	**<10**
**10**	**453**	**363**	**471**	**398**	**15 (450 nm)**	**<1**
**11**	**453**	**360**	**452**	**379**	**88 (405 nm)**	**<1**

The comparison between the ML-predicted electronic absorption bands and the experimental data shown in Table 2 clearly highlights the strength and utility of our model in identifying photoswitchable molecules with red-shifted and energetically separated π–π* transitions. However, it should be highlighted that several of the switches display low PSS compositions of the metastable isomer at the irradiation wavelengths used; these low PSS values of the *Z* isomer are attributed to a degree of overlap of broad electronic transitions of the isomeric forms. We envisage that the composition of the *Z* isomer at the PSS can be increased by expanding our compiled dataset to consider the full-width-at-half-max (FWHM) of the electronic absorption bands. Moreover, the thermal half-lives of the switches shown in Table 2 are short, less than 1 min. This rapid thermal relaxation is to be expected for the push–pull type photoswitches the ML model predicted. Despite showing some possible applications for information transfer, we hope to include a consideration of the thermal half-life properties in future work. This will undoubtedly improve the suitability of the predicted switches for a given application. Taken together, we anticipate that the ML model detailed here will be of use for synthetic chemists working to design photoswitchable molecules with red-shifted absorption bands and hope to incorporate additional photophysical and photochemical considerations in the future.

## 7 Conclusions

We have proposed a data-driven prediction pipeline underpinned by dataset curation and multioutput Gaussian processes. We demonstrated that a MOGP model trained on a small curated azophotoswitch dataset can achieve comparable predictive accuracy to TD-DFT and only slightly reduced performance relative to TD-DFT with a data-driven linear correction in near-instantaneous time. We use our methodology to discover several motifs that displayed separated electronic absorption bands of their isomers, as well as exhibiting a red-shifted absorption, and are suited for information transfer materials and towards photopharmacological applications. Sources of future work include the curation of an experimental dataset of the thermal reversion barriers to improve the predictive capabilities of machine learning models. Such a dataset would complement recent advances in machine learning prediction of thermal reversion barriers using quantum chemical photoswitch datasets,^82^ as well as machine learning approaches for accelerating the speed of quantum chemical simulations themselves.^83^ A further point of interest would be an investigation into how synthetic chemists may use model uncertainty estimates in the decision process to screen molecules *e.g. via* active learning^84^ and Bayesian optimisation. The confidence–error curves in the ESI† show initial promise in this direction and indeed understanding how best to tailor calibrated Bayesian models to molecular representations^65,85^ is an avenue worthy of pursuit. We release our curated dataset and all code to train models at https://github.com/Ryan-Rhys/The-Photoswitch-Dataset in order that the photoswitch community may derive benefit from our work.

## Data availability

All code, models and data are made available open-source at https://github.com/Ryan-Rhys/The-Photoswitch-Dataset.

## Author contributions

In terms of project conceptualisation, R.-R. G., A. R. T. and A. A. L. jointly initiated the project. A. R. T. suggested the prediction of transition wavelengths as a figure of merit. A. A. L. suggested the curation of a dataset and A. R. T. proposed the collated properties (Section 2). R.-R. G. proposed the idea of using Gaussian processes as the predictive model (Section 3). A. R. T. proposed the idea of an experimental comparison against TD-DFT and suggested a suitable reference paper (Section 4 and Section C6). R.-R. G. proposed the human performance comparison (Section 5 and Section C4). A. A. L. proposed the extension to screen for novel photoswitches using the machine learning model (Section 6). R.-R. G. proposed the idea of visualising photoswitch representations for which A. B. proposed the use of the UMAP algorithm (Section B). R.-R. G. proposed the machine learning benchmark (Section C.1). R.-R. G. proposed the fragprints representation (Section C.2). R.-R. G. proposed the out-of-domain generalisation experiment (Section C.3). R.-R. G. proposed the analysis of the Gaussian process confidence–error curves (Section C.5). A. A. L. proposed the assessment of diversity based on Tanimoto similarity (Section F). In terms of the project implementation, A. R. T. and R.-R. G. curated a dataset of molecular photoswitch properties from the literature (Section 2). R.-R. G. carried out the machine learning model benchmark study (Section 3), implementing code and running experiments for the random forest, Gaussian process, multioutput Gaussian process, Bayesian neural network, and SMILES-X models (Section C1). AJ implemented code for the GAT, GCN and MPNN models for which R.-R. G. ran the experiments (Section C1). P. J. implemented the code and ran experiments for the ANP model (Section C1). H. M. implemented code and ran experiments for the string kernel and SELFIES GP models (Section C1). W. M. implemented code and ran experiments for the directed message-passing neural network and the SOAP Gaussian process (Section C1). R.-R. G. aggregated all results and conducted the analysis of the machine learning model benchmark, including the Wilcoxon signed rank test for the efficacy of multitask learning (Section C1) R.-R. G. introduced the fragprints representation which yielded the best predictive performance on the benchmark (Section C2). R.-R. G. carried out the TD-DFT performance comparison experiments including the correlation and error distribution analysis (Section 4 and Section C6). A. R. T. devised and recruited participants for the human performance comparison (Section 5). R.-R. G. ran the MOGP model for the human performance comparison (Section 5). R.-R. G. conducted the out-of-domain generalisation experiment (Section C.3). A. A. L. generated the list of purchasable photoswitch molecules containing a diazo motif (Section 6). A. R. T. stated the criteria and suggested the scaffold (Section 6). R.-R. G. wrote all scripts for screening using the MOGP model, generating the list of lead candidates (Section 6). J. L. G. planned and conducted all experimental measurements including UV-vis spectroscopy and photoswitching. J. L. G. processed, fitted, assigned and interpreted all experimental data. J. L. G. and M. J. F. provided expertise in contextualising the predicted electronic properties of the photoswitches (Section 6). R.-R. G. devised and conducted the confidence–error curve experiments (Section C.5). R.-R. G. performed the data visualisation (Section B). In terms of writing, J. L. G. and R.-R. G. wrote the abstract. J. L. G. and R.-R. G. jointly wrote Section 1. J. L. G., A. R. T and R.-R. G. wrote Section 2. R.-R. G. wrote Section 3. R.-R. G. wrote Section 4. A. R. T. and R.-R. G. wrote Section 5. J. G. and R.-R. G. wrote Section 6. R.-R. G. and J. L. G. wrote Section 7. A. R. T. wrote Section A of the ESI.† R.-R. G. wrote Section B and C. A. A. wrote Section D. J. L. G. and R.-R. G. wrote Section E. R.-R. G. wrote Section F. A. R. T. and R.-R. G. wrote Section G. All authors reviewed the completed draft. A. A. L. and M. J. F. oversaw supervision and obtained funding for the study.

## Conflicts of interest

There are no conflicts to declare.

## Supplementary Material

SC-013-D2SC04306H-s001
