# Supplementary material for: Data-driven discovery of molecular photoswitches with multioutput Gaussian processes
Source: Chem Sci. 2022 Nov 10;13(45):13541–51. doi: 10.1039/d2sc04306h (PMC9682911; doi:10.1039/d2sc04306h)
Supplement: SC-013-D2SC04306H-s001 [file SC-013-D2SC04306H-s001.pdf]

Electronic Supplementary Material (ESI) for Chemical Science.
This journal is © The Royal Society of Chemistry 2022

ESI updated 16/11/2022

Supporting Information

Ryan-Rhys Griffiths,[*,a] Jake Greenfield,[b,c] Aditya R. Thawani,[b] Arian R. Jamasb,[d] Henry B. Moss,[e] Anthony Bourached,[f] Penelope Jones,[a] William McCorkindale,[a] Alexander Aldrick,[a] Matthew J. Fuchter,[b] and Alpha A. Lee[a]

a The Cavendish Laboratory, Department of Physics, University of Cambridge, Cambridge CB3 0HE, United Kingdom

b Molecular Sciences Research Hub, Department of Chemistry, Imperial College London, London W12 0BZ, United Kingdom

c Center for Nanosystems Chemistry (CNC), Universität Würzburg, Würzburg 97074, Germany; Institut für Organische Chemie, Universität Würzburg, Würzburg 97074, Germany

d The Computer Laboratory, University of Cambridge, Cambridge CB3 0FD, United Kingdom

e Secondmind.ai, Cambridge CB2 1LA, United Kingdom

f The Institute of Neurology, Department of Neurology, University College London, London WC1N 3BG, United Kingdom

E-mail: rrg27@cam.ac.uk

A Sources of Experimental Data

Properties were collated from a wide range of photoswitch literature. An emphasis was placed on collating compounds with a spectrum of functional groups attached to the core photoswitch scaffold. In addition, this dataset is unique in that it is composed of the latest generations of azoheteroarenes and cyclic azobenzenes which possess far superior photoswitch properties to analogous, unmodified azobenzenes. See Figure 1 for an overview of these novel azophotoswitches with their properties summarised.[1–22]

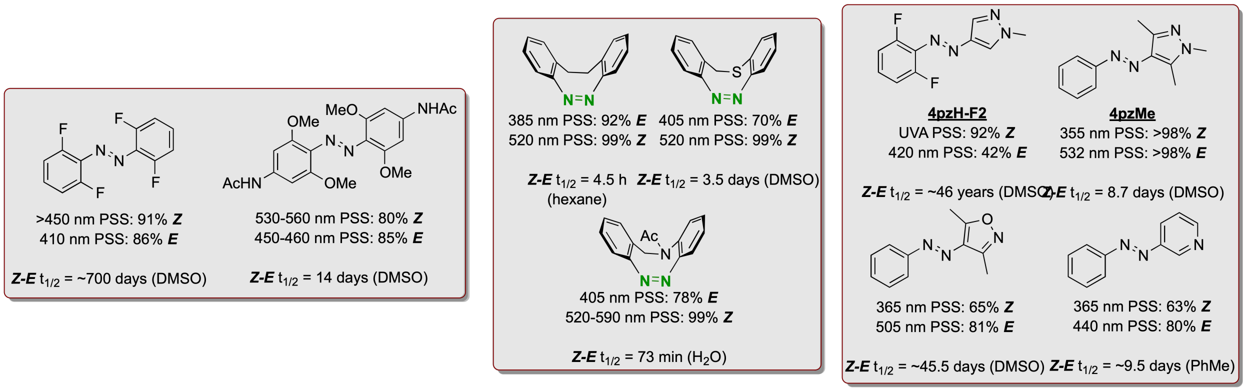

Figure 1: A data summary for the latest generation of azophotoswitches contained in this dataset. **PSS** = photostationary state, ***Z-E*** $t_{1/2}$ = Z isomer thermal half-life.

B Dataset Visualisations

The choice of molecular representation is known to be a key factor in the performance of machine learning algorithms on molecules.[23–25] Commonly-used representations such as fingerprint and fragment-based descriptors are high dimensional and as such, it can be challenging to interpret the inductive bias introduced by the representation. In order to visualise the high-dimensional representation space of the Photoswitch Dataset we project the data matrix to two dimensions using the UMAP algorithm.[26] We compare the manifolds located under the Morgan fingerprint representation and a fragment-based representation computed using RDKit.[27] We generate 512-bit Morgan fingerprints with a bond radius of 2, setting the nearest

neighbours parameter in the UMAP algorithm to a value of 50. The resulting visualisation was produced using the ASAP package (available at https://github.com/BingqingCheng/ASAP) and is shown in Figure 2.

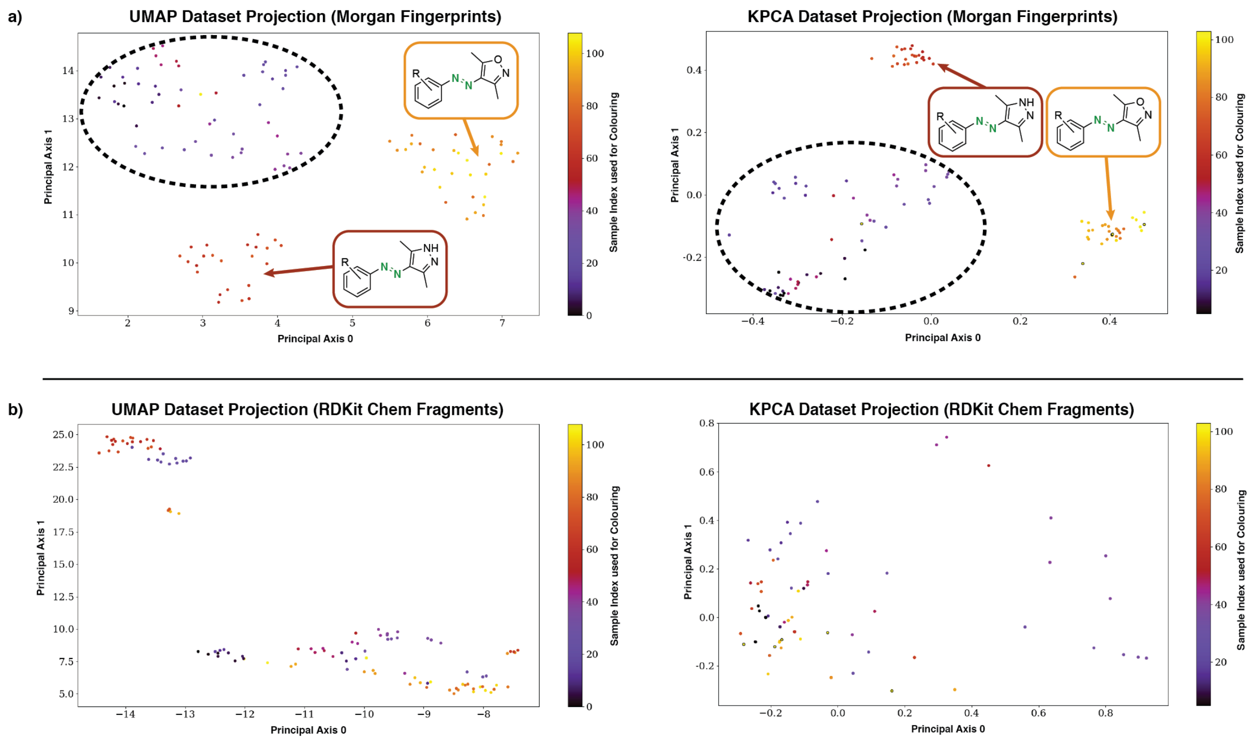

Figure 2: a) UMAP and k-PCA projections of the dataset, using Morgan Fingerprints, correctly identify clusters of chemically similar molecules. The regions demarcated by dashed black lines are composed of miscellaneous azoheteroarenes; no grouping was noted here due to the limited ($\leq$ 10) examples per class. b) Similar projections using RDKit Fragment descriptors fails to identify any such clusters.

The structure of the manifold located under the Morgan fingerprint representation identifies meaningful subgroups of azophotoswitches when compared to the fragment-based representation. In order to demonstrate that the finding is due to the representation and not the dimensionality reduction algorithm we include the manifolds identified by k-PCA using a cosine kernel. Both algorithms identify the same manifold structure in the Morgan fingerprint representation.

C Further Experiments

C.1 Property Prediction

For representations, we use 2048-bit Morgan fingerprints with a bond radius of 3 implemented in RDKit.[27] We use 85-dimensional fragment features computed using the RDKit descriptors module. We use the Dscribe library[28] to compute (Smooth Overlap of Atomic Positions) (SOAP) descriptors using an `rcut` parameter of 3.0, a `sigma` value of 0.2, an `nmax` parameter of 12 and an `lmax` parameter of 8. We use an REMatch kernel with polynomial base kernel of degree 3.0, `gamma` = 1.0, `coef0` = 0, `alpha` = 0.5 and `threshold` = $1e^{-6}$.

We evaluate performance on 20 random train/test splits in a ratio of 80/20 using the root mean square error (RMSE), mean absolute error (MAE) and coefficient of determination (R^2) as performance metrics, reporting the mean and standard error for each metric (Table 1). We evaluate the following models: Random Forest (RF), Gaussian Processes (GP), Attentive Neural Processes (ANP) ,[29] Graph Convolutional Networks (GCN),[30] Graph Attention Networks (GAT),[31] Directed Message-Passing Neural Networks (DMPNN),[32] and the following representations: Morgan fingerprints,[33] RDKit fragments,[27] SOAP,[34] the simplified molecular-input line-Entry system (SMILES),[35] and self-referencing embedded strings (SELFIES).[36] In addition, we introduce a hybrid representation, fragprints formed by concatenating the fragment and fingerprint vectors. For the purpose of the benchmark, hyperparameter selection for GP-based approaches is performed by optimizing the marginal likelihood on the train set whereas for other methods cross-validation is performed using the Hyperopt-Sklearn library[37] for Sklearn models such as RF and 1000 randomly sampled configurations for other models.

RF is trained using scikit-learn[38] with 1000 estimators and a maximum depth of 300. We implement a GP in GPflow[39] using a Tanimoto kernel[40,41] for fingerprint, fragment and fragprint representations, and the subset string kernel of[42] (following the exact experimental setup in Moss and Griffiths[41]) for the character-based SMILES and SELFIES representations.

Additionally, we train a multioutput Gaussian process (MOGP) based on the intrinsic model of coregionalisation[43] in order to leverage information in the multitask setting. For all GP models, we set the mean function to be the empirical mean of the data and treat the kernel variance and likelihood variance as hyperparameters, optimising their values under the marginal likelihood. For the attentive neural process we use 2 hidden layers of dimension 32 for each of the decoder, latent decoder and the deterministic encoder respectively, 8-dimensional latent variables r and z, and run 500 iterations with the Adam optimiser[44] with a learning rate of 0.001. For the ANP we perform principal components regression by reducing the representation dimension to 50. We implement GCNs and GATs in the DGL-LifeSci library.[45] Node features include one-hot representations of atom-type, atom degree, the number of implicit hydrogen atoms attached to each atom, the total number of hydrogen atoms per atom, atom hybridization, the formal charge and number of radical electrons on the atom. Edge features contain one-hot encodings of bond-type and Booleans indicating the stereogenic configuration of the bond and whether the bond is conjugated or in a ring. For the GCN we use two hidden layers with 32 hidden units and ReLU activations, applying BatchNorm[46] to both layers. For the GAT we use two hidden layers with 32 units each, 4 attention heads, an alpha value of 0.2 in both layers and ELU activations. We use a single DMPNN model trained for 50 epochs, with additional normalised 2D RDKit features. All remaining parameters were set to the default values in Yang et al.[32]. We do not benchmark SchNet[47] because it is designed for the prediction of molecular energies and atomic forces. All experiments were performed on the CPU of a MacBook Pro using a 2.3 GHz 8-Core Intel Core i9 processor.

We apply standardisation (subtract the mean and divide by the standard deviation) to the property values in all experiments. The results of the aforementioned models and representations are given in Table 1. Additional results including Message-passing neural networks (MPNN),[48] a black-box alpha divergence minimization Bayesian neural network (BNN),[49] and an LSTM with augmented SMILES, SMILES-X[50] are presented in Table 2.

We note that featurisations using standard molecular descriptors are more than competitive with neural representations for this dataset. The best-performing representation/model pair on the most data-rich E isomer $\pi - \pi^*$ task was the MOGP*-Tanimoto kernel and our own hybrid descriptor set "fragprints". Importantly, there is weak evidence that the MOGP* is able to leverage multitask learning in learning correlations between the transition wavelengths of the isomers, a modelling feature that may be particularly useful in the low-data regimes characteristic of experimental datasets. A Wilcoxon signed-rank test [51] is carried out in order to determine whether the performance differential between the GP/fragprints combination and the MOGP*/fragprints combination is statistically significant. In this instance, the MOGP* is provided with auxiliary task labels for test molecules where available (i.e. labels for tasks that are not being predicted). The null hypothesis is that there is no significant difference arising from multitask learning. In the case of the E isomer $\pi - \pi^*$ transition the resultant p-value is 0.33 meaning that we cannot reject the null hypothesis at the 95% confidence level. In the case of the Z isomer $\pi - \pi^*$ transition the resultant p-value is 0.06 meaning also that we cannot reject the null hypothesis at the 95% confidence level. In this latter case however, rejection of the null hypothesis depends on the confidence level threshold specified. As such, we conclude that only weak evidence is available to support the benefits of multitask learning over single task learning.

In this section we present, in Table 2 results with additional models on the property prediction benchmark for which extensive hyperparameter tuning was not undertaken. The black-box alpha divergence minimization Bayesian neural network is implemented in the Theano library [52] and is based on the implementation of. [49] the network has 2 hidden layers of size 25 with ReLU activations. The alpha parameter is set to 0.5, the prior variance for the variational distribution q is set to 1 and 100 samples are taken to approximate the expectation over the variational distribution. For all tasks the network is trained using 8 iterations of the Adam optimiser [44] with a batch size of 32 and a learning rate of 0.05. The MPNN is trained for 100 epochs in the case of the E isomer $\pi - \pi^*$ task and 200 epochs in

Table 1: Test set performance in predicting the transition wavelengths of the E and Z isomers. Best-performing models are highlighted in bold. MOGP* denotes a multioutput GP such that auxiliary task labels (i.e. not the task being predicted) for test molecules are provided to the model where available.

	E isomer $\pi-\pi^*$ (nm)	E isomer $n-\pi^*$ (nm)	Z isomer $\pi-\pi^*$ (nm)	Z isomer $n-\pi^*$ (nm)
**RMSE**				
RF + Morgan	25.3 ± 0.9	**10.2 ± 0.4**	14.0 ± 0.6	11.1 ± 0.4
RF + Fragments	26.4 ± 1.1	11.4 ± 0.5	17.0 ± 0.8	14.2 ± 0.6
RF + Fragprints	23.4 ± 0.9	11.0 ± 0.4	14.2 ± 0.6	11.3 ± 0.6
GP + Morgan	23.4 ± 0.8	11.4 ± 0.5	13.2 ± 0.7	**11.0 ± 0.7**
GP + Fragments	26.3 ± 0.8	11.6 ± 0.5	15.5 ± 0.8	12.6 ± 0.5
GP + Fragprints	20.9 ± 0.7	11.1 ± 0.5	13.1 ± 0.6	11.4 ± 0.7
GP + SOAP	21.0 ± 0.6	22.7 ± 0.6	17.8 ± 0.8	15.0 ± 0.5
GP + SMILES	26.0 ± 0.8	12.3 ± 0.4	12.5 ± 0.5	11.8 ± 0.6
GP + SELFIES	23.5 ± 0.7	12.9 ± 0.5	14.4 ± 0.5	12.2 ± 0.5
MOGP + Morgan	23.6 ± 0.8	11.7 ± 0.5	15.5 ± 0.6	11.1 ± 0.7
MOGP + Fragments	27.0 ± 0.9	11.9 ± 0.6	16.4 ± 0.9	13.1 ± 0.6
MOGP + Fragprints	21.2 ± 0.7	11.3 ± 0.5	13.5 ± 0.6	11.4 ± 0.7
MOGP* + Morgan	22.6 ± 0.8	11.6 ± 0.4	12.3 ± 0.7	10.9 ± 0.7
MOGP* + Fragments	26.9 ± 0.8	12.1 ± 0.6	16.2 ± 0.8	13.8 ± 0.6
MOGP* + Fragprints	**20.4 ± 0.7**	11.2 ± 0.5	**11.3 ± 0.4**	11.4 ± 0.7
ANP + Morgan	28.1 ± 1.3	13.6 ± 0.5	13.5 ± 0.6	**11.0 ± 0.6**
ANP + Fragments	27.9 ± 1.1	13.8 ± 0.9	17.2 ± 0.8	14.1 ± 0.7
ANP + Fragprints	27.0 ± 0.8	11.6 ± 0.5	14.5 ± 0.8	11.3 ± 0.7
GCN	22.0 ± 0.8	12.8 ± 0.8	16.3 ± 0.8	13.1 ± 0.8
GAT	26.4 ± 1.1	16.9 ± 1.9	19.6 ± 1.0	14.5 ± 0.8
DMPNN	27.1 ± 1.4	13.9 ± 0.6	17.5 ± 0.7	13.8 ± 0.4
**MAE**				
RF + Morgan	15.5 ± 0.5	**7.3 ± 0.3**	10.1 ± 0.4	**6.6 ± 0.3**
RF + Fragments	16.4 ± 0.5	8.5 ± 0.3	12.2 ± 0.6	9.0 ± 0.4
RF + Fragprints	13.9 ± 0.4	7.7 ± 0.3	10.0 ± 0.4	6.8 ± 0.3
GP + Morgan	15.2 ± 0.4	8.4 ± 0.3	9.8 ± 0.4	6.9 ± 0.3
GP + Fragments	17.3 ± 0.4	8.6 ± 0.3	11.5 ± 0.5	8.2 ± 0.3
GP + Fragprints	13.3 ± 0.3	8.2 ± 0.3	9.8 ± 0.4	7.1 ± 0.3
GP + SOAP	14.3 ± 0.3	19.3 ± 0.5	12.9 ± 0.6	11.4 ± 0.4
GP + SMILES	16.6 ± 0.5	8.6 ± 0.3	9.4 ± 0.4	7.4 ± 0.3
GP + SELFIES	14.7 ± 0.7	8.8 ± 0.3	11.1 ± 0.3	8.1 ± 0.2
MOGP + Morgan	15.3 ± 0.4	8.6 ± 0.3	11.9 ± 0.5	7.0 ± 0.3
MOGP + Fragments	17.6 ± 0.5	8.8 ± 0.4	12.1 ± 0.6	8.3 ± 0.3
MOGP + Fragprints	13.5 ± 0.3	8.3 ± 0.3	10.2 ± 0.5	7.1 ± 0.3
MOGP* + Morgan	14.4 ± 0.4	8.5 ± 0.3	9.6 ± 0.4	6.9 ± 0.4
MOGP* + Fragments	17.2 ± 0.4	8.9 ± 0.3	11.9 ± 0.5	8.5 ± 0.4
MOGP* + Fragprints	**13.1 ± 0.3**	8.3 ± 0.3	**8.8 ± 0.3**	7.1 ± 0.4
ANP + Morgan	17.9 ± 0.7	10.1 ± 0.4	10.0 ± 0.4	7.2 ± 0.3
ANP + Fragments	17.4 ± 0.6	9.4 ± 0.4	12.3 ± 0.6	8.9 ± 0.4
ANP + Fragprints	18.1 ± 0.5	8.6 ± 0.3	10.4 ± 0.5	7.0 ± 0.3
GCN	13.9 ± 0.3	8.6 ± 0.3	11.6 ± 0.5	8.6 ± 0.5
GAT	18.1 ± 0.7	10.7 ± 0.6	14.4 ± 0.8	10.8 ± 0.7
DMPNN	17.1 ± 0.8	10.6 ± 0.4	12.8 ± 0.6	9.8 ± 0.3
**R^2**				
RF + Morgan	0.85 ± 0.01	**0.80 ± 0.01**	0.25 ± 0.06	0.36 ± 0.06
RF + Fragments	0.83 ± 0.01	0.75 ± 0.02	−0.15 ± 0.11	−0.05 ± 0.07
RF + Fragprints	0.87 ± 0.01	0.77 ± 0.02	0.23 ± 0.07	0.33 ± 0.06
GP + Morgan	0.87 ± 0.01	0.76 ± 0.01	0.34 ± 0.05	**0.38 ± 0.05**
GP + Fragments	0.84 ± 0.01	0.74 ± 0.02	0.07 ± 0.08	0.19 ± 0.05
GP + Fragprints	**0.90 ± 0.01**	0.77 ± 0.02	0.35 ± 0.05	0.33 ± 0.05
GP + SOAP	0.89 ± 0.01	−0.08 ± 0.03	−0.05 ± 0.02	−0.07 ± 0.02
GP + SMILES	0.84 ± 0.02	0.72 ± 0.02	0.39 ± 0.05	0.29 ± 0.04
GP + SELFIES	0.86 ± 0.01	0.68 ± 0.02	0.20 ± 0.05	0.23 ± 0.04
MOGP + Morgan	0.87 ± 0.01	0.75 ± 0.01	0.06 ± 0.08	0.37 ± 0.05
MOGP + Fragments	0.83 ± 0.01	0.73 ± 0.02	−0.05 ± 0.10	0.11 ± 0.06
MOGP + Fragprints	0.89 ± 0.01	0.76 ± 0.02	0.30 ± 0.06	0.33 ± 0.05
MOGP* + Morgan	0.88 ± 0.01	0.75 ± 0.01	0.34 ± 0.12	0.39 ± 0.05
MOGP* + Fragments	0.83 ± 0.01	0.72 ± 0.02	−0.06 ± 0.12	0.00 ± 0.08
MOGP* + Fragprints	**0.90 ± 0.01**	0.76 ± 0.01	**0.49 ± 0.05**	0.33 ± 0.06
ANP + Morgan	0.70 ± 0.02	0.66 ± 0.02	0.30 ± 0.06	**0.38 ± 0.05**
ANP + Fragments	0.81 ± 0.01	0.62 ± 0.05	−0.16 ± 0.11	−0.06 ± 0.10
ANP + Fragprints	0.83 ± 0.01	0.75 ± 0.01	0.18 ± 0.08	0.35 ± 0.05
GCN	0.87 ± 0.01	0.66 ± 0.03	−0.41 ± 0.22	−0.92 ± 0.3
GAT	0.81 ± 0.02	0.57 ± 0.04	0.39 ± 0.17	−1.07 ± 0.4
DMPNN	0.82 ± 0.02	0.63 ± 0.02	−0.05 ± 0.07	0.11 ± 0.04

the case of the other tasks with a learning rate of 0.001 and a batch size of 32. The model architecture was taken to be the library default with the same node and edge features used for the GCN and GAT models in the main paper. The SMILES-X implementation remains the same as that of the paper[50] save for the difference that the network is trained for 40 epochs without Bayesian optimization over model architectures. In the case of SMILES-X 3 random train/test splits are used instead of 20 for the Z isomer tasks whereas 2 splits are used for the E isomer $n{-}\pi^*$ task. For the E isomer $\pi - \pi^*$ prediction task results are missing due to insufficient RAM on the machine used to run the experiments.

Table 2: Test set performance in predicting the transition wavelengths of the E and Z isomers.

	E isomer $\pi - \pi^*$ (nm)	E isomer $n{-}\pi^*$ (nm)	Z isomer $\pi - \pi^*$ (nm)	Z isomer $n{-}\pi^*$ (nm)
**RMSE**				
BNN + Morgan	27.0 ± 0.9	12.9 ± 0.6	13.9 ± 0.6	12.7 ± 0.4
BNN + Fragments	31.2 ± 1.1	14.8 ± 0.8	16.9 ± 0.8	12.7 ± 0.4
BNN + Fragprints	26.7 ± 0.8	13.1 ± 0.5	14.9 ± 0.5	13.0 ± 0.6
MPNN	24.8 ± 0.8	12.5 ± 0.6	16.7 ± 0.8	12.8 ± 0.7
SMILES-X		25.1 ± 4.2	17.8 ± 0.6	14.8 ± 0.9
**MAE**				
BNN + Morgan	19.0 ± 0.6	9.9 ± 0.4	10.2 ± 0.5	8.6 ± 0.3
BNN + Fragments	22.4 ± 0.8	10.6 ± 0.4	12.9 ± 0.6	8.6 ± 0.3
BNN + Fragprints	19.1 ± 0.6	10.1 ± 0.5	10.8 ± 0.4	9.3 ± 0.5
MPNN	15.4 ± 0.8	8.6 ± 0.3	11.6 ± 0.6	8.4 ± 0.4
SMILES-X		20.6 ± 3.1	11.6 ± 1.0	11.2 ± 1.0
R^2				
BNN + Morgan	0.83 ± 0.01	0.69 ± 0.02	0.23 ± 0.08	0.18 ± 0.05
BNN + Fragments	0.77 ± 0.01	0.58 ± 0.04	−0.15 ± 0.14	0.18 ± 0.05
BNN + Fragprints	0.83 ± 0.01	0.68 ± 0.02	0.14 ± 0.06	0.11 ± 0.08
MPNN	0.83 ± 0.01	0.63 ± 0.06	−0.70 ± 0.34	−0.68 ± 0.27
SMILES-X		−0.44 ± 0.30	−0.08 ± 0.06	−0.09 ± 0.04

C.2 Prediction Error as a Guide to Representation Selection

On the E isomer $\pi - \pi^*$ transition wavelength prediction task, we note occasionally marked discrepancies in the predictions made under the Morgan fingerprint and fragment representations. We show one such discrepancy in Figure 3. The resultant analysis motivated the expansion of the molecular feature set to include both representations as "fragprints"

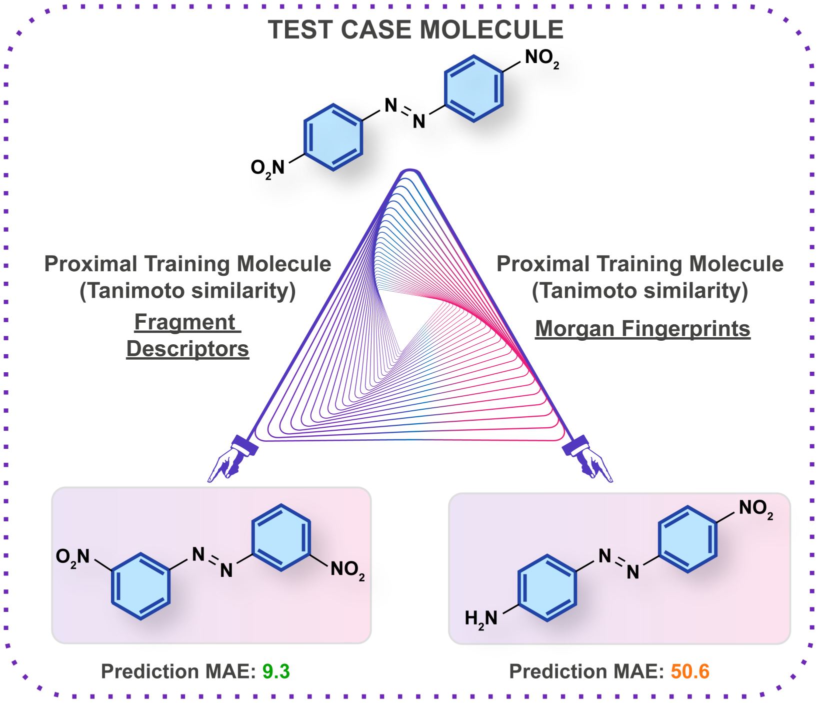

Figure 3: An analysis of the prediction errors under the Morgan fingerprint and fragment representations. The molecule on which the prediction is being made is located at the apex of the triangle with the proximal training molecule at the base. Fragment descriptors identify another di-substituted nitro-azobenzene as the most similar molecule contained in the train set. By contrast, Morgan fingerprints identify a molecule in possession of a similar substitution pattern to the test case, but with different functionalization. On this particular test instance it is the identity of the functional groups rather than the substitution pattern which dictates the wavelength properties and hence fragment descriptors achieve a much lower error. As such, although fingerprints offer better overall performance, fragments are clearly informative features for certain test cases.

C.3 Impact of Dataset Choice

In this section we evaluate the generalisation performance of a model trained on the E isomer $\pi - \pi^*$ values of a large dataset of 6142 out-of-domain molecules (including non-azoarene photoswitches) from Beard et al.[53] with experimentally-determined labels. We train a Random Forest regressor (due to scalability issues with the MOGP on 6000+ data points) implemented in the scikit-learn library with 1000 estimators and a max depth of 300 on the fragprint representation of the molecules. In Table 3 we present results for the case when the train set consists of the large dataset of 6142 molecules and the test set consists of the entire photoswitch dataset. We also present the results on the original E isomer $\pi - \pi^*$ transition wavelength prediction task where the train set of each random 80/20 train/test split is augmented with the molecules from the large dataset. The results indicate that the data for out-of-domain molecules provides no benefit for the prediction task and even degrades performance, when amalgamated, relative to training on in-domain data only.

Table 3: Performance comparison of curated dataset against large non-curated dataset.

Dataset	Size	RMSE	MAE	R^2
Large Non-Curated	6142	85.2	72.5	−0.66
Large Non-Curated + Curated	6469	36.9 ± 1.2	22.7 ± 0.7	0.67 ± 0.02
Curated	314	$\mathbf{23.4 \pm 0.9}$	$\mathbf{13.9 \pm 0.4}$	$\mathbf{0.87 \pm 0.01}$

Based on these results we highlight the importance of designing synthetic molecular machine learning benchmarks with a real-world application in mind and involving synthetic chemists in the curation process. By targeted data collation on a narrow and well-defined region of chemical space where the molecules are in-domain relative to the task, it becomes possible to mitigate generalisation error.

C.4 Human Performance Benchmark

Below in Table 4 we include the full results breakdown of the human performance comparison study.

Table 4: Results breakdown for the human expert performance comparison predicting the transition wavelength (nm) of the E isomer $\pi - \pi^*$ transition for 5 molecules. Closest prediction for each molecule is underlined and highlighted in bold. MOGP achieves the lowest MAE relative to all individual human participants.

	Molecule 1	Molecule 2	Molecule 3	Molecule 4	Molecule 5	MAE
True Value	**329**	**407**	**333**	**540**	**565**	
Postdoc 1	325	360	410	490	490	54.7
PhD 1	350	400	530	410	425	93.3
PhD 2	380	280	530	600	250	177.5
Postdoc 2	<u>**330**</u>	350	500	475	500	66.7
PhD 3	325	350	350	<u>**540**</u>	550	16.3
Postdoc 3	350	370	520	600	500	97.5
PhD 4	<u>**330**</u>	380	390	520	580	34.2
Undergraduate 1	340	420	400	<u>**540**</u>	570	41.8
Postdoc 4	321	345	<u>**340**</u>	500	520	28.7
PhD 5	<u>**330**</u>	360	<u>**340**</u>	500	520	24.2
PhD 6	303	367	435	411	450	78.7
PhD 7	280	350	450	430	460	85.5
PhD 8	270	390	420	420	440	73.8
PhD 9	<u>**330**</u>	310	462	512	512	55.3
MOGP	321	<u>**413**</u>	354	518	<u>**569**</u>	<u>**11.9**</u>

C.5 Confidence-Error Curves

An advantage of Bayesian models for the real-world prediction task is the ability to produce calibrated uncertainty estimates. If correlated with prediction error, a model's uncertainty may act as an additional decision-making criterion for the selection of candidates for lab synthesis. In order to investigate the benefits afforded by uncertainty estimates, we produce confidence-error curves using the GP-Tanimoto model in conjunction with the fingerprints representation. The confidence-error curves for the RMSE and MAE metrics are shown in Figure 4 and Figure 5 respectively. The x-axis, confidence percentile, may be obtained simply by ranking each model prediction of the test set in terms of the predictive variance at the location of that test input. As an example, molecules that lie in the 80th confidence percentile will be the 20% of test set molecules with the lowest model uncertainty. We then measure the prediction error at each confidence percentile across 200 random train/test splits to see whether the model's confidence is correlated with the prediction error. We observe that across all tasks the GP-Tanimoto model's uncertainty estimates are positively correlated with prediction error, offering a proof of concept that model uncertainty can be incorporated into the decision process for candidate selection.

C.6 TD-DFT Benchmark

Below, in Figure 6 and Figure 7 we include further plots analysing the performance of the methods on the TD-DFT performance comparison benchmark. These plots motivated the use of the Lasso-correction to the TD-DFT predictions.

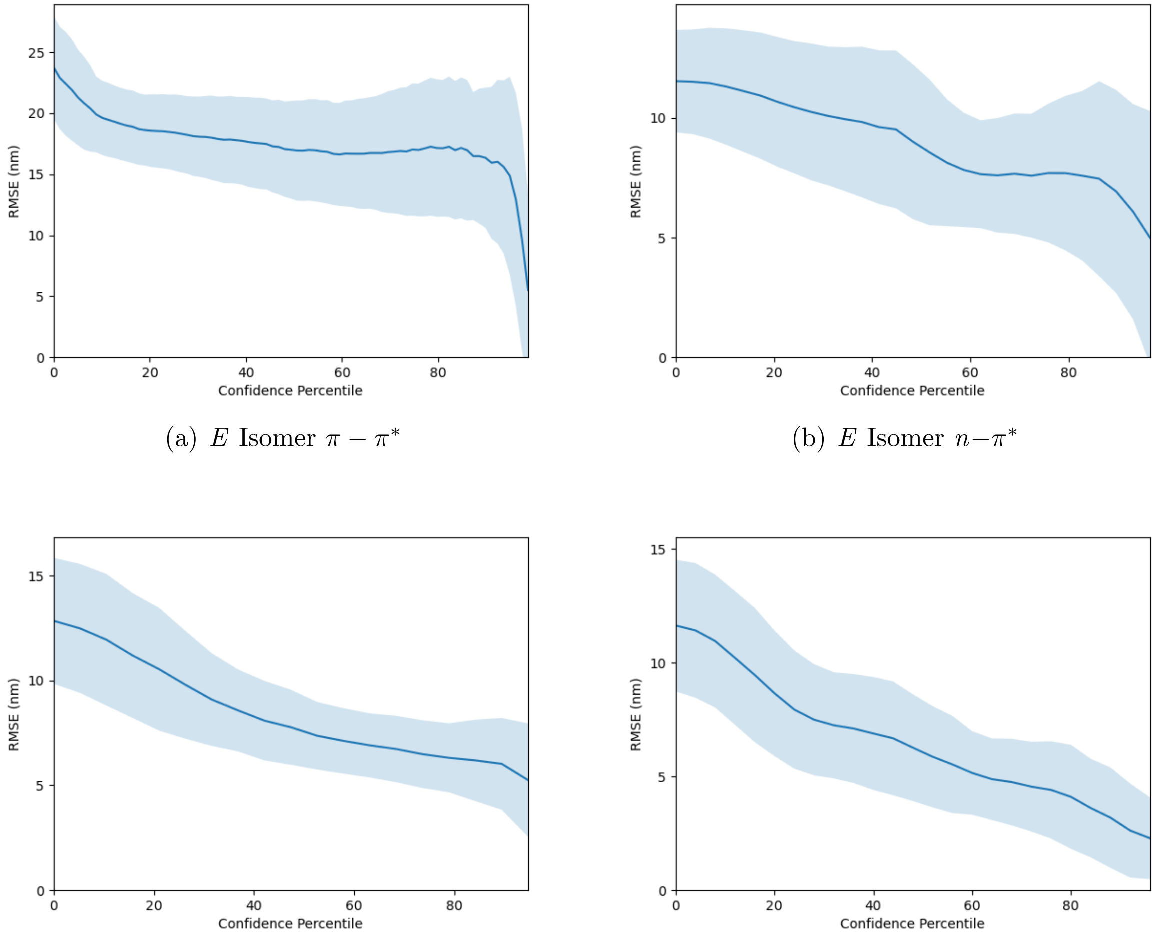

(a) E Isomer $\pi - \pi^*$

(b) E Isomer n–π^*

(c) Z Isomer $\pi - \pi^*$

(d) Z Isomer n–π^*

Figure 4: RMSE Confidence-Error Curves for Property Prediction using GP Regression.

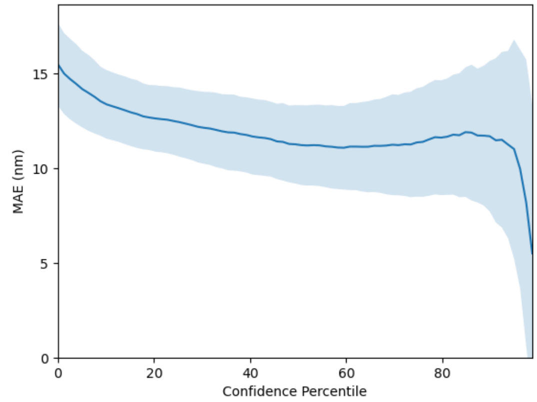

(a) E Isomer $\pi - \pi^*$

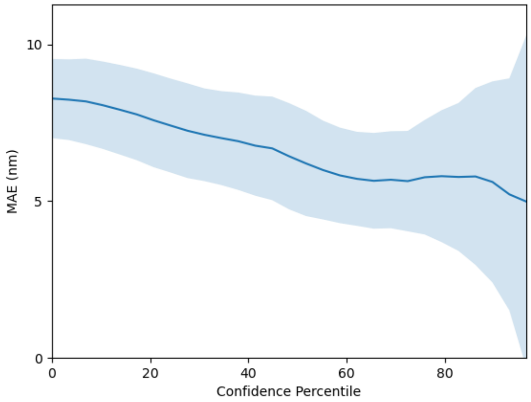

(b) E Isomer n–π^*

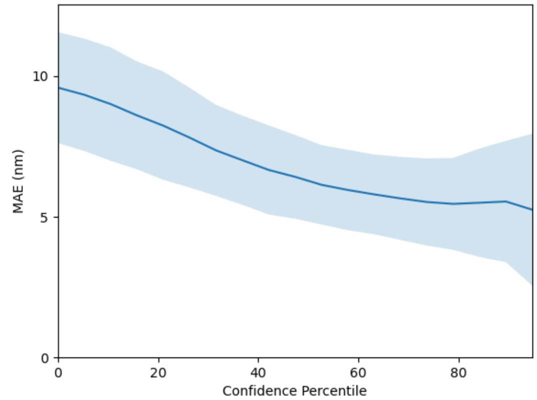

(c) Z Isomer $\pi - \pi^*$

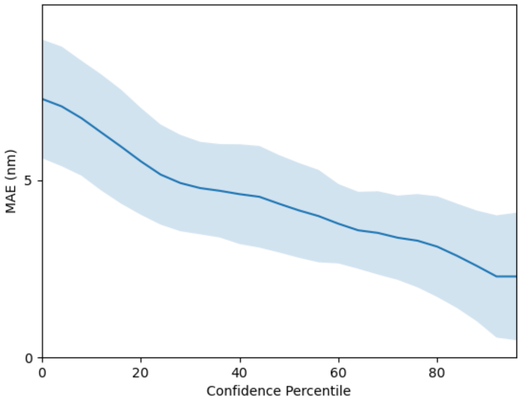

(d) Z Isomer n–π^*

Figure 5: MAE Confidence-Error Curves for Property Prediction using GP Regression.

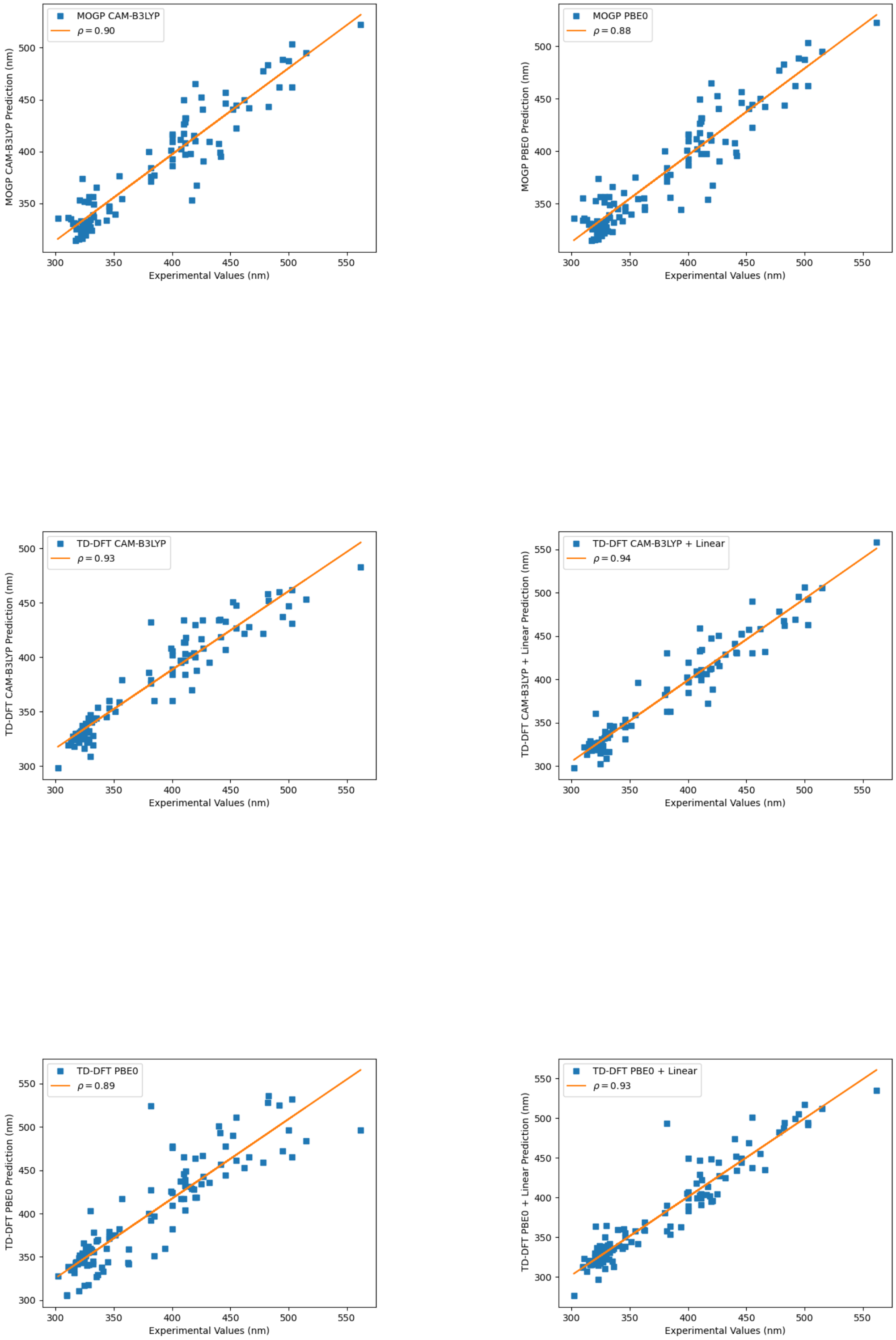

Figure 6: Regression plots for each method on the TD-DFT performance comparison benchmark with the Spearman rank-order correlation coefficient given as ρ. One may observe that the correlation between predictions and ground truth experimental values increases with the linear Lasso correction to the TD-DFT methods.

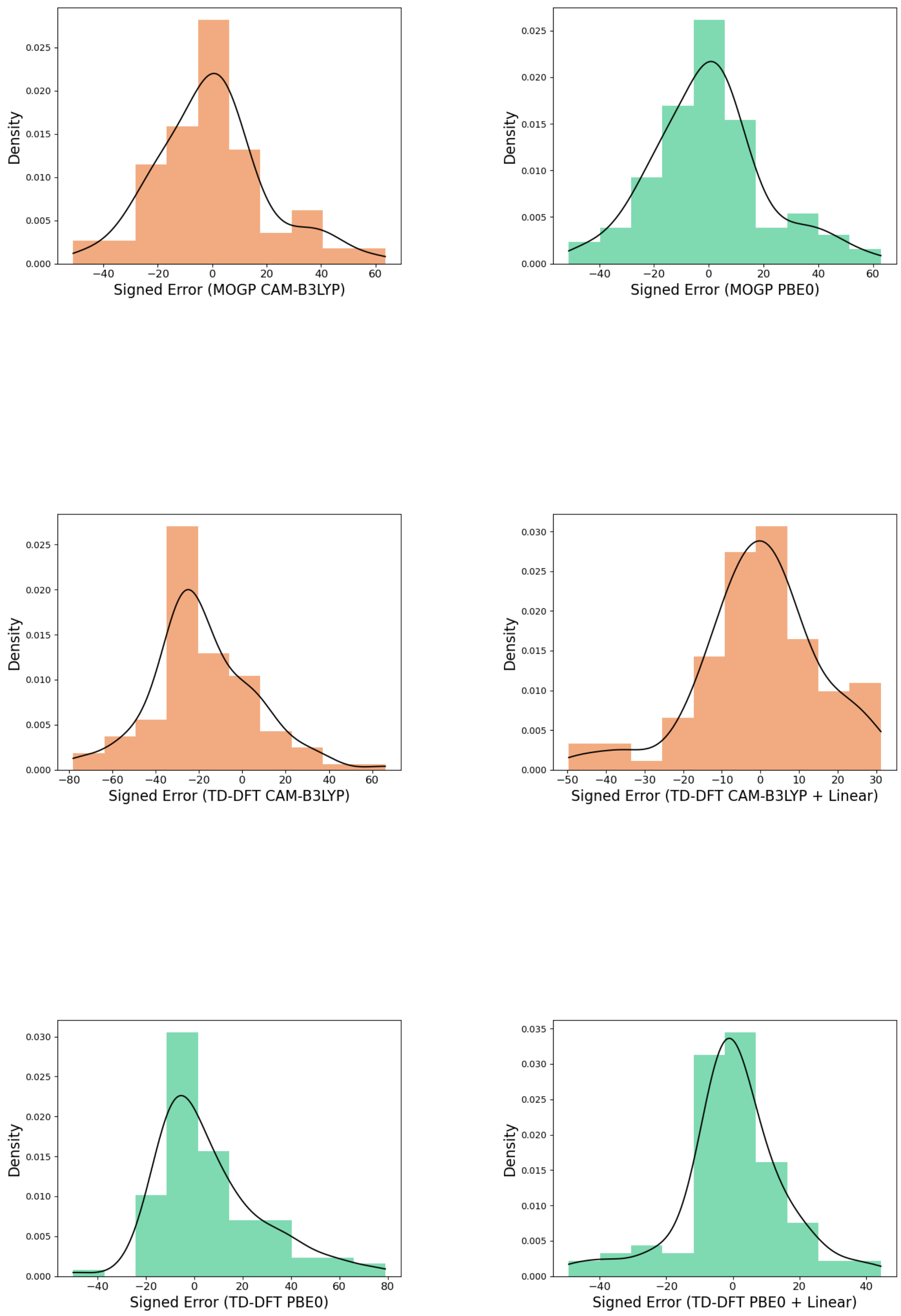

Figure 7: Signed error distributions for each method on the TD-DFT performance comparison benchmark. Signed error is recorded for each heldout molecule in leave-one-out-validation. Gaussian kernel density estimates overlaid on the histograms. One may observe that the linear Lasso correction for the TD-DFT methods has a centering effect on the error distribution.

D Background on Time-Dependent Density Functional Theory

D.1 Density Functional Theory

Density Functional Theory (DFT) is a modelling method used to elucidate the electronic structure (typically the ground state) of many-body systems.[54] The theory has been used with great success across physics, chemistry, biology and materials science.[55] DFT is considered to be an *ab initio*, or first principles method because it relies directly upon the postulates of quantum mechanics and the only inputs to the calculations are physical constants.[56] A concrete example of an application of DFT towards an electronic structure investigation is in simulating a relaxation of atoms in a crystalline solid to calculate the change in lattice parameters and the forces on each atom, with the introduction of defects or vacancies into the system.[57]

Since its inception in 1964/5, Kohn-Sham DFT (KS-DFT) has been one of the most popular electronic structure methods to date.[55] KS-DFT relies on the Hohenberg-Kohn theorems[58] and the use of a trial electron density (an initial guess) with a self-consistency scheme. In practice, a computational loop takes a trial density, solves the Kohn-Sham equations, and obtains the single electron wavefunctions corresponding to the trial density; next, by taking these single electron wavefunctions and using a result of quantum mechanics, a calculated electron density can be computed. If this calculated density is consistent (within a set tolerance) of the trial density, then the theoretical ground state density has been found. If the two densities are not consistent, the calculated density is taken as the new trial density, and the loop is repeated until the tolerance is met. With exchange and correlation functionals, the accuracy of DFT calculations can be very high, but may also fluctuate significantly with the choice of functional, pseudopotential, basis sets and cutoff energy[59] which are not always straightforward to optimise. A machine learning corollary would be the performance of a specific model, on a given dataset, greatly depending on its hyperparameters, with

out-of-the-box implementations rarely giving satisfactory results without a significant amount of tuning.

D.2 Time-Dependent Density Functional Theory

Time-dependent Density Functional Theory (TD-DFT) is based on a time-dependent cognate of the Hohenberg-Kohn theorems; the Runge-Gross (RG) theorem.[60] This theorem shows that a unique delineation exists between the time-dependent electron density and the time-dependent external potential. This allows for a simplification, permitting a computational time-dependent Kohn-Sham system to be substantiated[61] analogous to the computational system used in KS-DFT.

In conjunction with a linear response theory,[62] TD-DFT has excelled with investigations into calculating electromagnetic spectra, i.e. absorption spectra, of medium and large molecules.[63,64] It has become popular in these fields, due to its ease of use relative to other methods as well as its high accuracy. A relevant application of this methodology is to compute the $\pi - \pi^*/n - \pi^*$ electronic transitions wavelengths for conjugated molecular systems, such as the photoswitch molecules in the Photoswitch Dataset.

E Further Screening Details

The SMILES for all experimentally-measured molecules are given in Table 5. Reagents and solvents were obtained from commercial sources (MolPort) and used as supplied.

E.1 UV-Vis Absorption Spectroscopy

UV-Vis absorption spectra were obtained on an Agilent 8453 UV-Visible Spectrophotometer G1103A. A sampler holder with four open faces was used to enable in-situ irradiation (90° to the measurement beam). Samples were prepared in a UV Quartz cuvette with a path length of 10 mm. Solutions of the compounds were prepared in HPLC grade DMSO at a

concentration of 25 μM.

E.2 Photoswitching

Samples were irradiated with a custom-built irradiation set up using 365 nm (3 × 800 mW Nichia NCSU276A LEDs, FWHM 9 nm), 405 nm (3 × 770 mW Nichia NCSU119C LEDs, FWHM 11 nm), 450 nm (3 × 900 mW Nichia NCSC219B-V1 LEDs, FWHM 18 nm), 495 nm (3 × 750 mW Nichia NCSE219B-V1 LEDs, FWHM 32 nm), 525 nm (3 × 450 mW NCSG219B-V1 LEDs, FWHM 38 nm) and 630 nm (3 × 780 mW Nichia NCSR219B-V1 LEDs, FWHM 16 nm) light sources. Samples were irradiated until no further change in the UV-vis absorption spectra was observed, indicating that the Photostationary State (PSS) was reached.

The PSS, and the "predicted pure Z" spectra was determined using UV-vis following the procedure reported by Fischer[65].

Table 5: ID and SMILES strings for the 11 lead candidates identified for lab measurement.

ID	SMILES
1	CCOC1=CC=C(/N=N/C2=C3N=CC(C#N)=C(N)N3N=C2N)C=C1
2	NC1=NN2C(N)=C(C#N)C=NC2=C1/N=N/C3=CC4=C(OCO4)C=C3
3	COC1=CC=C(C2=NC3=C(C(N)=NN3C(N)=C2C#N)/N=N/C4=CC5=C(OCO5)C=C4)C=C1
4	O=S(C1=CC=C(/N=N/C2=C3N=C(C4=CC=CS4)C(C#N)=C(N)N3N=C2N)C=C1)(N)=O
5	CSC1=C(C(N)=NC2=C(C(N)=NN12)/N=N/C3=CC=C(S(N)(=O)=O)C=C3)C#N
6	COC1=CC=C(/N=N/C2=C3N=C(C4=CC=CC=C4)C(C#N)=C(N)N3N=C2N)C=C1
7	[O-]Cl(=O)(=O)=O.CCN(C1=CC=C(/N=N/C2=C([N+]([O-])=O)C(C)=[N+](N2C)C)C=C1)CC
8	CN(C1=CC=C(/N=N/C2=NC(C#N)=C(C#N)N2)C=C1)C
9	CN1C(/N=N/C2=CC=C(NC3=CC=CC=C3)C=C2)=NC4=CC=CC=C14
10	CCN(S(=O)(C1=CC=C(/N=N/C2=C(C3=CC=CC=C3)N=C(N)S2)C=C1)=O)CC
11	O=C1N(C2=CC=CC=C2)N(C)C(C)=C1/N=N/C3=CC=C(N)C(OC)=C3

F Novelty of Screened Candidates relative to The Photoswitch Dataset

In Figure 8, for each of the 6 candidates satisfying both performance criteria, we give some indication as to the novelty of the discovered photoswitch candidates by providing the 3 closest molecules by Tanimoto similarity from the photoswitch dataset.

G Datasheets for Datasets

Following the protocol outlined in[66] we provide a datasheet for our dataset of key transition wavelengths for azophotoswitches that we release as part of this submission:

G.1 Motivation

For what purpose was the dataset created? Was there a specific task in mind? Was there a specific gap that needed to be filled? Please provide a description.

The primary objective of this dataset is to assimilate a list of experimentally determined electronic transition wavelengths describing the key $\pi - \pi^*$ and $n - \pi^*$ electronic transitions of photoswitchable molecules. At present, there is no dataset that addresses the needs of synthetic chemists who would ideally like to know the aforementioned properties of a photoswitch prior to synthesising it so as to minimise the amount of synthetic effort required. In an imaginary, and grossly simplified work-flow, a synthetic chemist may be tasked with red-shifting the $\pi - \pi^*$ transition wavelength of an initial hit molecule for a given biological application. Faced with countless possible modifications that could be made to the initial hit, it is likely that a significant effort could be expended synthesising molecules that do not achieve the desired objective. In an ideal

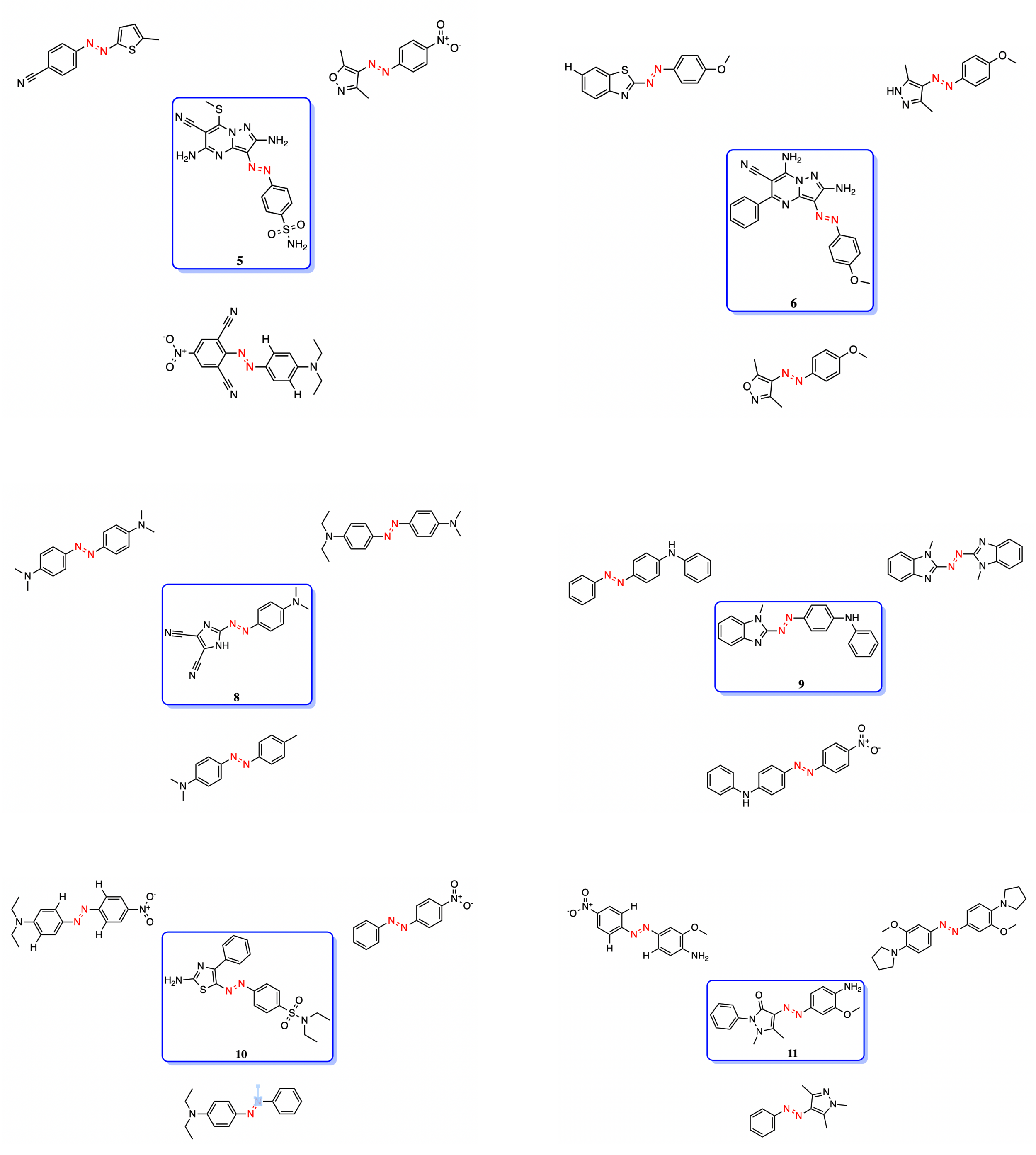

Figure 8: All 6 experimentally-tested candidates satisfying both performance criteria together with the 3 closest molecules by Tanimoto similarity in the photoswitch dataset.

world, said chemist would utilise TD-DFT to predict the spectral properties of each and every molecule prior to synthesis, however, as described in the attached manuscript, this is a slow process. Thus, the central aim of this dataset is to act as a catalyst for developing machine learning models that accurately predict the spectral properties of azophotoswitches in an efficient manner and with accuracy comparable to the state-of-the-art TD-DFT methods. The main manuscript describes how this aim has been achieved to a certain degree.

Who created this dataset (e.g., which team, research group) and on behalf of which entity (e.g., company, institution, organization)?

The dataset was manually curated in 2020 by Dr. Aditya Raymond Thawani whilst pursuing a PhD at the Department of Chemistry, Imperial College London.

Who funded the creation of the dataset? If there is an associated grant, please provide the name of the grantor and the grant name and number.

No financial support was necessary or received in pursuit of the current work.

G.2 Composition

What do the instances that comprise the dataset represent (e.g., documents, photos, people, countries)? Are there multiple types of instances (e.g., movies, users, and ratings; people and interactions between them; nodes and edges)? Please provide a description. How many instances are there in total (of each type, if appropriate)?

Throughout this discussion the reader is reminded that each azophotoswitch

molecule has two isomeric forms i.e. *E* and *Z*. The dataset assimilates experimentally determined characteristics of a series of azophotoswitches. The dataset includes molecular properties for 405 photoswitch molecules. All molecular structures are denoted according to the simplified molecular input line entry system (SMILES). We collate the following properties for the molecules:

Rate of Thermal Isomerization (units of s^{-1}): This is a measure of the thermal stability of the least stable isomer (*Z* isomer for non-cyclic azophotoswitches and *E* isomer for cyclic azophotoswitches). Measurements are carried out in solution with the compounds dissolved in the stated solvents.

Photostationary State (units = % of stated isomer): Upon continuous irradiation of an azophotoswitch a steady state distribution of the *E* and *Z* isomers is achieved. Measurements are carried out in solution with the compounds dissolved in the 'irradiation solvents'.

pi-pi*/n-pi* wavelength (units = nanometers): The wavelength at which the pi-pi*/n-pi* electronic transition has a maxima for the stated isomer. Measurements are carried out in solution with the compounds dissolved in the 'irradiation solvents'.

DFT-computed pi-pi*/n-pi* wavelengths (units = nanometers): DFT-computed wavelengths at which the pi-pi*/n-pi* electronic transition has a maxima for the stated isomer.

Extinction coefficient: The molar extinction coefficient.

Wiberg Index: A measure of the bond order of the N=N bond in an azophotoswitch. Bond order is a measure of the 'strength' of said bond. This value is computed theoretically through the analysis of the SCF density calculated at the PBE0/6-31G level of theory**

Irradiation wavelength: The specific wavelength of light used to irradiate samples from *E*-*Z* or *Z*-*E* such that a photo stationary state is obtained. Measurements are carried out in solution with the compounds dissolved in the 'irradiation solvents'.

Does the dataset contain all possible instances or is it a sample (not necessarily random) of instances from a larger set? If the dataset is a sample, then what is the larger set? Is the sample representative of the larger set (e.g., geographic coverage)? If so, please describe how this representativeness was validated/verified. If it is not representative of the larger set, please describe why not (e.g., to cover a more diverse range of instances, because instances were withheld or unavailable).

This dataset is intended to act as a reasonable sample of relevant azophotoswitches from the literature with a focus on ensuring diversity i.e. inclusion of more modern azoheteroarenes, cyclic azobenzenes and azobenzenes with a wide ranging diversity of functional groups (electron withdrawing and donating) appended on to either or both phenyl rings . Experimental data was collated from 21 literature references. No bias was shown in selecting molecules for inclusion in the dataset, however, compounds with experimental data of inadequate quality (e.g. improper experimental setups or missing values) were excluded. No tests were run to determine representativeness.

Is any information missing from individual instances? If so, please provide a description, explaining why this information is missing (e.g., because it was unavailable). This does not include intentionally removed information, but might include, e.g., redacted text.

No data has been intentionally excluded. The absence of any data is indicative of the fact that said data was not experimentally determined for the given molecule.

Are there recommended data splits (e.g., training, development/validation, testing)? If so, please provide a description of these splits, explaining the rationale behind them.

Scaffold splits and chronological splits are not as important in the case of experimental synthetic chemistry. We are not attempting to produce a model that achieves predictive accuracy for useful experimental properties across a large region of chemical space nor would we entertain this notion as being chemically realistic.

Are there any errors, sources of noise, or redundancies in the dataset? If so, please provide a description.

The irradiation solvent in which an experimental measurement was taken may act as a source of noise in our model formulation. Given that the transition wavelengths of the molecules in the dataset were measured under different irradiation solvents, we may either treat the choice of irradiation solvent as an additional categorical input feature to the model or we can absorb the choice of irradiation solvent into the measurement noise. We elected to take the latter modelling approach due its practicality in light of missing data on irradiation

solvents for some molecules in the dataset. Explicitly modelling the irradiation solvent choice as an input feature would either require excluding many data points, limiting the size of the training set, or it would require imputation of the unobserved solvent variable. Imputation methods include substituting the most frequent solvent in the dataset as the "true" irradiation solvent which would not be formally correct and may bias the model, or training a separate model to predict the missing solvent categories which would be highly challenging if not impossible due to the lack of training data. As such, we adopted the most consistent formulation of treating the choice of irradiation solvent as a latent (unobserved) variable that contributes to the transition wavelength value but as a source of measurement noise rather than as a component of the representation of the photoswitch molecules.

Is the dataset self-contained, or does it link to or otherwise rely on external resources (e.g., websites, tweets, other datasets)? If it links to or relies on external resources, a) are there guarantees that they will exist, and remain constant, over time; b) are there official archival versions of the complete dataset (i.e., including the external resources as they existed at the time the dataset was created); c) are there any restrictions (e.g., licenses, fees) associated with any of the external resources that might apply to a future user? Please provide descriptions of all external resources and any restrictions associated with them, as well as links or other access points, as appropriate.

All data has been extracted from internationally recognised and renowned chemical journals. The dataset itself does not rely on access to or link to these journals or associated papers. References for the papers from which said data has been extracted are provided.

G.3 Collection

How was the data associated with each instance acquired? Was the data directly observable (e.g., raw text, movie ratings), reported by subjects (e.g., survey responses), or indirectly inferred/derived from other data (e.g., part-of-speech tags, model-based guesses for age or language)? If data was reported by subjects or indirectly inferred/derived from other data, was the data validated/verified? If so, please describe how.

The data was observed as raw text in the aforementioned journal articles and manually transcribed from said journal articles to the stated dataset.

Has an analysis of the potential impact of the dataset and its use on data subjects (e.g., a data protection impact analysis)been conducted? If so, please provide a description of this analysis, including the outcomes, as well as a link or other access point to any supporting documentation.

Not applicable.

G.4 Preprocessing/cleaning/labelling

Not applicable.

G.5 Uses

Has the dataset been used for any tasks already? If so, please provide a description.

The dataset was used to predict electronic transitions of a range of syntheti-

cally accessible azophotoswitches to gauge their properties and decide on which compounds to synthesise. A candidate molecule was identified with promising spectral properties, synthesised and experimentally characterised. The experimental characterisation validated the initial prediction. Further work is ongoing to deploy this compound in a photopharmacological application.

Is there a repository that links to any or all papers or systems that use the dataset? If so, please provide a link or other access point.

The repository is hosted at https://github.com/Ryan-Rhys/The-Photoswitch-Dataset

What (other) tasks could the dataset be used for?

The dataset may be used to predict the thermal isomerisation properties of azophotoswitches given that thermal half-life data is tabulated within said dataset.

Is there anything about the composition of the dataset or the way it was collected and preprocessed/cleaned/labeled that might impact future uses? For example, is there anything that a future user might need to know to avoid uses that could result in unfair treatment of individuals or groups (e.g., stereotyping, quality of service issues) or other undesirable harms (e.g., financial harms, legal risks) If so, please provide a description. Is there anything a future user could do to mitigate these undesirable harms?

No risks involved or possibility of any harm: the data is contained within publicly available journal articles.

Are there tasks for which the dataset should not be used? If so, please provide a description.

There are no associated issues with using this dataset for other tasks. However, clearly when dealing with the chemical synthesis of any molecules indicated in this dataset due care should be shown along with a full safety and risk assessment.

G.6 Distribution

Will the dataset be distributed to third parties outside of the entity (e.g., company, institution, organization) on behalf of which the dataset was created? If so, please provide a description.

The dataset is publicly available on the internet.

How will the dataset will be distributed (e.g., tarball on website, API, GitHub)? Does the dataset have a digital object identifier (DOI)?

The dataset is hosted on GitHub. The DOI is https://zenodo.org/badge/latestdoi/232307189

When will the dataset be distributed?

The dataset was first released in June 2020.

Will the dataset be distributed under a copyright or other intellectual property (IP) license, and/or under applicable terms of use (ToU)? If so, please describe this license and/or ToU, and provide a link or other access point to, or otherwise reproduce, any relevant licensing terms or ToU, as well as any fees associated with these restrictions.

The crawled data copyright belongs to the authors of the reviews unless otherwise stated. There is no license, but there is a request to cite the corresponding paper if the dataset is used:

@articleThawani2020,

author = "Aditya Raymond Thawani, Ryan-Rhys Griffiths, Arian R. Jamasb et al.",

title = "The Photoswitch Dataset: A Molecular Machine Learning Benchmark for the Advancement of Synthetic Chemistry",

year = "2020",

month = "7",

doi = "10.26434/chemrxiv.12609899.v1"

G.7 Maintenance

Who is supporting/hosting/maintaining the dataset?

Aditya Raymond Thawani and Ryan-Rhys Griffiths.

How can the owner/curator/manager of the dataset be contacted (e.g., email address)?

art12@ic.ac.uk and rrg27@cam.ac.uk

Will the dataset be updated (e.g., to correct labeling errors, add new instances, delete instances)? If so, please describe how often, by whom, and how updates will be communicated to users (e.g., mailing list, GitHub)?

All relevant updates to the dataset will be publicised via GitHub and major revision via Twitter.

If others want to extend/augment/build on/contribute to the dataset, is there a mechanism for them to do so? If so, please provide a description. Will these contributions be validated/verified? If so, please describe how. If not, why not? Is there a process for communicating/distributing these contributions to other users? If so, please provide a description.

Interested parties should contact the original authors about incorporating updates.

G.8 Repository URL

The dataset repository is hosted at https://github.com/Ryan-Rhys/The-Photoswitch-Dataset.

G.9 Author Statement

We state, as authors that we bear all responsibility in case of violation of rights, etc., and confirmation of the data license.

G.10 Hosting, Licensing and Maintenance Plan

The dataset is hosted on GitHub under an MIT licence and will be maintained by Aditya Raymond Thawani and Ryan-Rhys Griffiths.

G.11 Repository DOI

The repository DOI is https://zenodo.org/badge/latestdoi/232307189

References

(1) Calbo, J.; Thawani, A. R.; Gibson, R. S.; White, A. J.; Fuchter, M. J. A combinatorial approach to improving the performance of azoarene photoswitches. *Beilstein Journal of Organic Chemistry* **2019**, *15*, 2753–2764.

(2) Calbo, J.; Weston, C. E.; White, A. J.; Rzepa, H. S.; Contreras-García, J.; Fuchter, M. J. Tuning azoheteroarene photoswitch performance through heteroaryl design. *Journal of the American Chemical Society* **2017**, *139*, 1261–1274.

(3) Weston, C. E.; Richardson, R. D.; Haycock, P. R.; White, A. J.; Fuchter, M. J. Arylazopyrazoles: azoheteroarene photoswitches offering quantitative isomerization and long thermal half-lives. *Journal of the American Chemical Society* **2014**, *136*, 11878–11881.

(4) Siewertsen, R.; Neumann, H.; Buchheim-Stehn, B.; Herges, R.; Nather, C.; Renth, F.; Temps, F. Highly efficient reversible Z- E photoisomerization of a bridged azobenzene with visible light through resolved S1 (npi*) absorption bands. *Journal of the American Chemical Society* **2009**, *131*, 15594–15595.

(5) Rustler, K.; Nitschke, P.; Zahnbrecher, S.; Zach, J.; Crespi, S.; Konig, B. Photochromic Evaluation of 3 (5)-Arylazo-1 H-pyrazoles. *The Journal of Organic Chemistry* **2020**, *85*, 4079–4088.

(6) Knie, C.; Utecht, M.; Zhao, F.; Kulla, H.; Kovalenko, S.; Brouwer, A. M.; Saalfrank, P.; Hecht, S.; Bléger, D. ortho-Fluoroazobenzenes: visible light switches with very long-lived Z isomers. *Chemistry–A European Journal* **2014**, *20*, 16492–16501.

(7) Sell, H.; Näther, C.; Herges, R. Amino-substituted diazocines as pincer-type photochromic switches. *Beilstein journal of organic chemistry* **2013**, *9*, 1–7.

(8) Thies, S.; Sell, H.; Bornholdt, C.; Schütt, C.; Köhler, F.; Tuczek, F.; Herges, R. Light-Driven Coordination-Induced Spin-State Switching: Rational Design of Photodissociable Ligands. *Chemistry–A European Journal* **2012**, *18*, 16358–16368.

(9) Devi, S.; Saraswat, M.; Grewal, S.; Venkataramani, S. Evaluation of Substituent Effect in Z-Isomer Stability of Arylazo-1 H-3, 5-dimethylpyrazoles: Interplay of Steric, Electronic Effects and Hydrogen Bonding. *The Journal of Organic Chemistry* **2018**, *83*, 4307–4322.

(10) Kumar, P.; Srivastava, A.; Sah, C.; Devi, S.; Venkataramani, S. Arylazo-3, 5-dimethylisoxazoles: Azoheteroarene Photoswitches Exhibiting High Z-Isomer Stability, Solid-State Photochromism, and Reversible Light-Induced Phase Transition. *Chemistry–A European Journal* **2019**, *25*, 11924–11932.

(11) Slavov, C.; Yang, C.; Heindl, A. H.; Wegner, H. A.; Dreuw, A.; Wachtveitl, J. Thiophenylazobenzene: An Alternative Photoisomerization Controlled by Lone-Pair pi Interaction. *Angewandte Chemie* **2020**, *132*, 388–395.

(12) Jacquemin, D.; Preat, J.; Perpète, E. A.; Vercauteren, D. P.; André, J.-M.; Ciofini, I.; Adamo, C. Absorption spectra of azobenzenes simulated with time-dependent density functional theory. *International Journal of Quantum Chemistry* **2011**, *111*, 4224–4240.

(13) Mustroph, H.; Gussmann, F. Studies on UV/Vis Absorption Spectra of Azo Dyes. 24. The different effect of a 2-methoxy and a 3-methoxy group in 4-NN-diethylaminoazobenzenes on colour. *Journal für Praktische Chemie* **1990**, *332*, 93–97.

(14) Mustroph, H. Studies on the UV-vis absorption spectra of azo dyes: Part 25. analysis of the fine structure of the pi1-pi1* band of 4-donor-sub. *Dyes and pigments* **1991**, *15*, 129–137.

(15) Mustroph, H. Studies on UV/Vis absorption spectra of azo dyes.: Part 26. electronic absorption spectra of 4, 4'-diaminoazobenzenes. *Dyes and pigments* **1991**, *16*, 223–230.

(16) Bridgeman, I.; Peters, A. Synthesis and Electronic Spectra of some 4–Aminoazobenzenes. *Journal of the Society of Dyers and Colourists* **1970**, *86*, 519–524.

(17) Seferoğlu, Z.; Ertan, N.; Hökelek, T.; Şahin, E. The synthesis, spectroscopic properties and crystal structure of novel, bis-hetarylazo disperse dyes. *Dyes and Pigments* **2008**, *77*, 614–625.

(18) Dinçalp, H.; Yavuz, S.; Haklı, Ö.; Zafer, C.; Özsoy, C.; Durucasu, İ.; İçli, S. Optical and photovoltaic properties of salicylaldimine-based azo ligands. *Journal of photochemistry and Photobiology A: Chemistry* **2010**, *210*, 8–16.

(19) Kennedy, A. D.; Sandler, I.; Andréasson, J.; Ho, J.; Beves, J. E. Visible-Light Photoswitching by Azobenzazoles. *Chemistry–A European Journal* **2020**, *26*, 1103–1110.

(20) Faustino, H.; Brannigan, C.; Reis, L.; Santos, P.; Almeida, P. Novel azobenzothiazole dyes from 2-nitrosobenzothiazoles. *Dyes and Pigments* **2009**, *83*, 88–94.

(21) Saylam, A.; Seferoğlu, Z.; Ertan, N. Azo-8-hydroxyquinoline dyes: the synthesis, characterizations and determination of tautomeric properties of some new phenyl-and heteroarylazo-8-hydroxyquinolines. *Journal of Molecular Liquids* **2014**, *195*, 267–276.

(22) Yen, M. S.; Wang, J. Synthesis and absorption spectra of hetarylazo dyes derived from coupler 4-aryl-3-cyano-2-aminothiophenes. *Dyes and Pigments* **2004**, *61*, 243–250.

(23) Faber, F. A.; Hutchison, L.; Huang, B.; Gilmer, J.; Schoenholz, S. S.; Dahl, G. E.; Vinyals, O.; Kearnes, S.; Riley, P. F.; Von Lilienfeld, O. A. Prediction errors of molecular machine learning models lower than hybrid DFT error. *Journal of Chemical Theory and Computation* **2017**, *13*, 5255–5264.

(24) Wu, Z.; Ramsundar, B.; N. Feinberg, E.; Gomes, J.; Geniesse, C.; S. Pappu, A.; Leswing, K.; Pande, V. MoleculeNet: A Benchmark for Molecular Machine Learning. *Chemical Science* **2018**, *9*, 513–530.

(25) Christensen, A. S.; Bratholm, L. A.; Faber, F. A.; Anatole von Lilienfeld, O. FCHL revisited: faster and more accurate quantum machine learning. *The Journal of Chemical Physics* **2020**, *152*, 044107.

(26) McInnes, L.; Healy, J.; Saul, N.; Großberger, L. UMAP: Uniform Manifold Approximation and Projection. *Journal of Open Source Software* **2018**, *3*, 861.

(27) Landrum, G., et al. RDKit: Open-source cheminformatics. **2006**,

(28) Himanen, L.; Jäger, M. O.; Morooka, E. V.; Canova, F. F.; Ranawat, Y. S.; Gao, D. Z.; Rinke, P.; Foster, A. S. DScribe: Library of descriptors for machine learning in materials science. *Computer Physics Communications* **2020**, *247*, 106949.

(29) Kim, H.; Mnih, A.; Schwarz, J.; Garnelo, M.; Eslami, A.; Rosenbaum, D.; Vinyals, O.; Teh, Y. W. Attentive Neural Processes. International Conference on Learning Representations. 2019.

(30) Kipf, T. N.; Welling, M. Semi-Supervised Classification with Graph Convolutional Networks. International Conference on Learning Representations (ICLR). 2017.

(31) Veličković, P.; Cucurull, G.; Casanova, A.; Romero, A.; Liò, P.; Bengio, Y. Graph Attention Networks. *In International Conference on Learning Representations* **2018**,

(32) Yang, K.; Swanson, K.; Jin, W.; Coley, C.; Eiden, P.; Gao, H.; Guzman-Perez, A.; Hopper, T.; Kelley, B.; Mathea, M., et al. Analyzing learned molecular representations for property prediction. *Journal of chemical information and modeling* **2019**, *59*, 3370–3388.

(33) Rogers, D.; Hahn, M. Extended-connectivity fingerprints. *Journal of chemical information and modeling* **2010**, *50*, 742–754.

(34) Bartók, A. P.; Kondor, R.; Csányi, G. On representing chemical environments. *Physical Review B* **2013**, *87*, 184115.

(35) Weininger, D. SMILES, a chemical language and information system. 1. Introduction to methodology and encoding rules. *Journal of chemical information and computer sciences* **1988**, *28*, 31–36.

(36) Krenn, M.; Häse, F.; Nigam, A.; Friederich, P.; Aspuru-Guzik, A. Self-Referencing Embedded Strings (SELFIES): A 100% robust molecular string representation. *Machine Learning: Science and Technology* **2020**, *1*, 045024.

(37) Komer, B.; Bergstra, J.; Eliasmith, C. *Automated Machine Learning*; Springer, Cham, 2019; pp 97–111.

(38) Pedregosa, F. et al. Scikit-learn: Machine Learning in Python. *Journal of Machine Learning Research* **2011**, *12*, 2825–2830.

(39) De G. Matthews, A. G.; Van Der Wilk, M.; Nickson, T.; Fujii, K.; Boukouvalas, A.; León-Villagrá, P.; Ghahramani, Z.; Hensman, J. GPflow: A Gaussian process library using TensorFlow. *The Journal of Machine Learning Research* **2017**, *18*, 1299–1304.

(40) Ralaivola, L.; Swamidass, S. J.; Saigo, H.; Baldi, P. Graph kernels for chemical informatics. *Neural networks* **2005**, *18*, 1093–1110.

(41) Moss, H. B.; Griffiths, R.-R. Gaussian process molecule property prediction with FlowMO. *arXiv preprint arXiv:2010.01118* **2020**,

(42) Moss, H.; Leslie, D.; Beck, D.; Gonzalez, J.; Rayson, P. BOSS: Bayesian Optimization over String Spaces. *Advances in Neural Information Processing Systems* **2020**, *33*.

(43) Williams, C.; Bonilla, E. V.; Chai, K. M. Multi-task Gaussian process prediction. *Advances in Neural Information Processing systems* **2007**, 153–160.

(44) Kingma, D.; Ba, J. Adam: A method for stochastic optimization. *arXiv preprint* **2014**,

(45) Wang, M. et al. Deep Graph Library: Towards Efficient and Scalable Deep Learning on Graphs. *ICLR Workshop on Representation Learning on Graphs and Manifolds* **2019**,

(46) Ioffe, S.; Szegedy, C. Batch Normalization: Accelerating Deep Network Training by Reducing Internal Covariate Shift. Proceedings of the 32nd International Conference on International Conference on Machine Learning - Volume 37. 2015; pp 448–456.

(47) Schütt, K.; Kindermans, P.-J.; Sauceda, H.; Chmiela, S.; Tkatchenko, A.; Müller, K.-R. SchNet: A continuous-filter convolutional neural network for modeling quantum interactions. Proceedings of the 31st International Conference on Neural Information Processing Systems. 2017; pp 992–1002.

(48) Gilmer, J.; Schoenholz, S. S.; Riley, P. F.; Vinyals, O.; Dahl, G. E. Neural message passing for quantum chemistry. Proceedings of the 34th International Conference on Machine Learning - Volume 70. 2017; pp 1263–1272.

(49) Hernández-Lobato, J. M.; Li, Y.; Rowland, M.; Hernández-Lobato, D.; Bui, T. D.; Turner, R. E. Black-box α-divergence minimization. Proceedings of the 33rd International Conference on International Conference on Machine Learning-Volume 48. 2016; pp 1511–1520.

(50) Lambard, G.; Gracheva, E. SMILES-X: Autonomous molecular compounds characterization for small datasets without descriptors. *Machine Learning: Science and Technology* **2020**, *1*, 025004.

(51) Wilcoxon, F. Individual Comparisons by Ranking Methods. *Biometrics Bulletin* **1945**, *1*, 80–83.

(52) Theano Development Team, Theano: A Python framework for fast computation of mathematical expressions. *arXiv e-prints* **2016**, *abs/1605.02688*.

(53) Beard, E. J.; Sivaraman, G.; Vázquez-Mayagoitia, Á.; Vishwanath, V.; Cole, J. M. Comparative dataset of experimental and computational attributes of UV/vis absorption spectra. *Scientific Data* **2019**, *6*, 1–11.

(54) Brázdová, V.; Bowler, D. R. *Atomistic computer simulations: a practical guide*; Wiley-VCH: Weinheim, 2013; OCLC: ocn835961914.

(55) Becke, A. D. Perspective: Fifty years of density-functional theory in chemical physics. *The Journal of Chemical Physics* **2014**, *140*, 18A301.

(56) Leach, A. R.; Leach, A. R. *Molecular modelling: principles and applications*; Pearson education, 2001.

(57) Hasnip, P. J.; Refson, K.; Probert, M. I.; Yates, J. R.; Clark, S. J.; Pickard, C. J. Density functional theory in the solid state. *Philosophical Transactions of the Royal Society A: Mathematical, Physical and Engineering Sciences* **2014**, *372*, 20130270.

(58) Hoenberg, P.; Kohn, W. Inhomogeneous electron gas. *Phys. Rev* **1964**, *136*, B864–B871.

(59) Howard, J. C.; Enyard, J. D.; Tschumper, G. S. Assessing the accuracy of some popular DFT methods for computing harmonic vibrational frequencies of water clusters. *The Journal of Chemical Physics* **2015**, *143*, 214103.

(60) Heinze, H. H.; Görling, A.; Rösch, N. An efficient method for calculating molecular excitation energies by time-dependent density-functional theory. *The Journal of Chemical Physics* **2000**, *113*, 2088–2099.

(61) van Leeuwen, R. Causality and symmetry in time-dependent density-functional theory. *Physical review letters* **1998**, *80*, 1280.

(62) Ullrich, C. A. *Time-dependent density-functional theory: concepts and applications*; OUP Oxford, 2011.

(63) Casida, M. E.; Huix-Rotllant, M. Progress in time-dependent density-functional theory. *Annual review of physical chemistry* **2012**, *63*, 287–323.

(64) Burke, K.; Werschnik, J.; Gross, E. Time-dependent density functional theory: Past, present, and future. *The Journal of Chemical Physics* **2005**, *123*, 062206.

(65) Fischer, E. Calculation of photostationary states in systems A $\rightleftharpoons$ B when only A is known. *J. Phys. Chem.* **1967**, *71*, 3704–3706.

(66) Gebru, T.; Morgenstern, J.; Vecchione, B.; Vaughan, J. W.; Wallach, H.; Daumé III, H.; Crawford, K. Datasheets for datasets. *arXiv preprint arXiv:1803.09010* **2018**,
